# Estimating heritability using family-pooled phenotypic and genotypic data: a simulation study applied to aquaculture

**DOI:** 10.1038/s41437-022-00502-8

**Published:** 2022-01-31

**Authors:** Nima Khalilisamani, Peter Campbell Thomson, Herman Willem Raadsma, Mehar Singh Khatkar

**Affiliations:** 1 Sydney School of Veterinary Science, Faculty of Science, The University of Sydney, Camden, NSW 2570 Australia; 2 ARC Research Hub for Advanced Prawn Breeding, James Cook University, Townsville, QLD 4811 Australia

**Keywords:** Genome evolution

## Abstract

Estimating heritability based on individual phenotypic and genotypic measurements can be expensive and labour-intensive in commercial aquaculture breeding. Here, the feasibility of estimating heritability using within-family means of phenotypes and allelic frequencies was investigated. Different numbers of full-sib families and family sizes across ten generations with phenotypic and genotypic information on 10 K SNPs were analysed in ten replicates. Three scenarios, representing differing numbers of pools per family (one, two and five) were considered. The results showed that using one pool per family did not reliably estimate the heritability of family means. Using simulation parameters appropriate for aquaculture, at least 200 families of 60 progeny per family divided equally in two pools per family was required to estimate the heritability of family means effectively. Although application of five pools generated more within- and between- family relationships, it reduced the number of individuals per pool and increased within-family residual variation, hence, decreased the heritability of family means. Moreover, increasing the size of pools resulted in increasing the heritability of family means towards one. In addition, heritability of family mean estimates were higher than family heritabilities obtained from Falconer’s formula due to lower intraclass correlation estimate compared to the coefficient of relationship.

## Introduction

Narrow-sense heritability determines the extent to which additive genetic component is contributing to the phenotypic variation among individuals or families (Viana 2002, Mathew et al. 2012, Kruijer et al. 2015, Kumar et al. 2016, Speed et al. 2017). Accurate heritability estimates are the foundation in estimating breeding values, selection of the superior broodstock to populate the next generation, and for calculating the genetic gain (Viana 2002, Mathew et al. 2012, Gay et al. 2013, Sun et al. 2015). Heritability can be estimated using information obtained either from pedigree or genotypic data. In traditional quantitative genetics, genetic parameters are usually estimated using information on the pedigree and the phenotypic values of individuals to estimate heritability (Bérénos et al. 2014, de los Campos et al. 2015). One popular approach for pedigree-based genetic analysis is Henderson’s linear mixed model (Henderson 1950), which only estimates the additive genetic component of the phenotypic variance. In the context of mixed model, Henderson has also introduced mixed model equation (MME), which can estimate additive genetic variance using fixed and random effects. However, MME can also estimate non-additive genetic components using pedigree and/or genotypic information. Nevertheless, using molecular markers to capture the relationship between individuals via construction of a genomic relationship matrix (GRM) can be more accurate compared to pedigree information (Gay et al. 2013, Bérénos et al. 2014, de los Campos et al. 2015, Kim et al. 2015, Kruijer et al. 2015), hence, can be more advantageous in estimating genetic components.

Using genotypic data and phenotypic measurements on individuals is a straightforward methodology for estimating genetic parameters, including heritability. However, this method might not be cost-effective approach in large-scale commercial breeding programs (Olson et al. 2006, Biscarini et al. 2010). For example, collecting phenotypic measurements, genotyping, and analysing data for body weight at harvest in a commercial fish farm at an individual level can be time consuming, labour intensive, expensive and difficult procedures, especially when the population size is large (Simianer and Gjerde 1991, Peeters et al. 2013). Consequently, it can be an expensive approach to gather genotypic or phenotypic data to estimate heritability for every trait using individual measurements (Olson et al. 2006, Su et al. 2018). In addition, individual genotypic or phenotypic values might not be available or recorded in every scenario. In some scenarios, analysis of data inferred from groups or pools of individuals can be a more feasible approach in estimating genetic parameters.

Using the average of phenotypic data from pools or groups of individuals is being practiced in breeding programs for estimating (co-)variance components and has been studied using simulation (Simianer and Gjerde 1991, Olson et al. 2006, Su et al. 2018) and empirical studies (Biscarini et al. 2008, Biscarini et al. 2010). These studies reported that the pooling strategy reduced the accuracy of estimated breeding values (EBVs). For example, in a simulation design on feed intake in pigs, Olson et al. (2006) demonstrated that when individuals were randomly assigned in pools of unequal sizes (between 12 and 24) comprised of two, three or more families, accuracy of EBVs inferred from fitting the linear mixed model for the pooled phenotypic data decreased as compared to individual EBV accuracies. In an empirical study on egg production in chicken, Biscarini et al. (2010) have also shown that when ~15,200 chickens from two batches were assigned in cages comprising of four individuals from either one or different families, the resulting EBVs obtained from pools were smaller than those inferred from individual measurements. However, heritability estimates obtained from pooled data were similar to individual-based estimations.

The above-mentioned studies have used only pedigree information. However, in several scenarios in aquaculture, the phenotype can only be recorded at the group or family level e.g., feed efficiency and survival traits. Moreover, the depth of the pedigree, for computing relationship between families, may not be available. In such scenarios, using genotypic and phenotypic data pooled at family level provide an attractive avenue.

The only study which applied both phenotype and genotype at pooled-data level, in a within-family context, to estimate heritability was conducted on ryegrass (Ashraf et al. 2016), which showed that construction of the GRM and estimation of family heritability is feasible using pooled data. The application of pooled data in aquaculture settings where often families are produced in large groups is more relevant. In addition, breeding design for several commercial species in aquaculture depends on family selection. When family selection is being practised, family means have a predictably smaller residual (environmental) variation. Therefore, mean of phenotypic variation come closer to the mean of genotypic variation (Falconer and Mackay 1996) and result in high heritability estimates. In this study we extended the approach used by Ashraf et al. (2016) for estimating the GRM and heritability from mean family phenotypic values and family mean allele frequencies in aquaculture using simulated data. We investigated the effect of different number of families, family sizes, number of pools per family and pooling size on estimation of heritability of family means. In addition, changes in heritability of family means across low, medium, and high trait heritability was also measured.

## Materials and methods

A number of scenarios and sub-scenarios were generated, representing different number of families, family sizes and trait heritabilities used to estimate the heritability of family means using both within-family pooled phenotypic and genotypic values.

### Simulation of populations

Simulation of pedigree and genotypic data was conducted using QMSim software (Sargolzaei and Schenkel 2009). Firstly, 400 historical generations with a constant population size of 2000 in each generation was used with 1000 males and 1000 females being generated in the last historical generation. From the last historical generation, different number of sires and dams were selected to form the base generation (G0) described in the following scenarios:

Scenario 1 (S1) consisted of eight different sub-scenarios representing 50, 100, 200 and 400 full-sib families, which were randomly sampled from the last historical generation. The generation of full-sib families was considered with inclusion of equal number of sires and dams, e.g., 50 sires and 50 dams in the 50 full-sib family sub-scenario. Each sub-scenario was assumed to produce 20 progeny per family per generation for ten discrete generations from generation 1 (G01) to generation 10 (G10). Selection of sires and dams was based on highest EBVs from generation one onwards whilst sires and dams were mated randomly.

Scenario 2 (S2) comprised four sub-scenarios with 100 and 200 full-sib families sampled randomly from the last historical generation, and each pair generated 40 or 60 progeny per family per generation from G01 to G10. The structure of full-sib families, selection and mating was the same as previously mentioned in S1.

Scenario 3 (S3) consisted of 50 full-sib families which were randomly sampled to form the last historical generation, with 200 progeny per family produced per generation from G01 to G10. Full-sib families were generated the same way as described in S1 whilst selection was based on highest EBVs and mating was at random as well.

Initially, each scenario and its associated sub-scenarios were generated considering a trait with a moderate heritability (*h*^2^: 0.3) with standardised mean of 0 and phenotypic variance of 1. In the absence of dominance and epistasis effects, phenotypic values were obtained from summing the random error, the polygenic effect, and the sum of QTL effects. To allow both QTL and polygenic effects to contribute to variation of the trait, the combined effect of all QTLs were sampled from normal distribution with µ = 0 and σ² = 0.2, allowing a third of the variance (0.1) to be attributed to the polygenic effect. Later, traits with low (0.05) and high (0.5) heritability were also generated for the sub-scenario with 200 families of 60 progeny each from S2 considering the same standardised mean and phenotypic variance. To assign as close as possible to a third of variation of traits to polygenic effect in the latter occasion, mean QTL effect (μ) was set to 0.03 and 0.3 for trait heritabilities of 0.05 and 0.5 respectively. Every scenario/sub-scenario was simulated in ten independent replicates. The summary of scenarios is provided in the Table 1.

**Table 1:** The structure of main scenario and sub-scenarios generated for estimating genetic parameters from family-pooled phenotypic and genotypic data.

			Heritability (*h²*)				
Scenario	No. of families	Family size	0.05	0.3	0.5	No. of replicates	No. of datasets
S1	50	20		✓		10	40
	100						
	200						
	400						
S2	100	40		✓		10	10
		60		✓			10
	200	40		✓			10
		60	✓	✓	✓		30
S3	50	200		✓		10	10
Total	–		–	–	–	–	110

The EBVs were calculated within QMSim software which uses Henderson’s method for the best linear unbiased predictions (Henderson 1975) incorporating the numerator relationship matrix (NRM) in every simulated generation.

### Genome structure

The genome was simulated with a similar structure in all scenarios/sub-scenarios consisting of 44 chromosomes of 50 cM each with 500 markers and 100 QTLs per chromosome. This generated 22,000 biallelic markers and 4400 biallelic QTLs per generation. Both markers and QTL were randomly positioned along the chromosome, sampled from a uniform distribution in each replicate and equal allele frequencies in the first historical generation. All loci in the first historical population had two alleles with a mutation rate of 10^−8^ per generation for markers and QTLs in the succeeding generations. Although linkage disequilibrium (LD) is not defined in the parameter file, the simulated mating design generated extensive LD across the genome across generations using minor allelic frequency (MAF) of higher than 0.05.

### Data preparation

The phenotypic means for each family were calculated by averaging the phenotypic values of individuals in each family in S1 and S2. This was simply considered as a single pool. In addition, in order to estimate the effect of pooling on heritability of family means, within each sub-scenario in S2, two and five pools per family were generated by randomly allocating family members equally across the pools. This resulted in 30 individuals per family per pool in two pools and 12 individuals per family per pool in the five-pool system in the 60 progeny per family scheme. Then, the mean of phenotypes was calculated for each pool. For example, in the 200-families scenario, there were 400 phenotypic means in the two-pool strategy and 1000 pools in the five-pool strategy. For S3, the same methodology was applied for generating only two pools but using different pool sizes (12, 30, 60 and 100 per pool). Consequently, in this scenario, totals of 24, 60, 120 and 200 progeny per family were used, randomly split into two equal pools.

Quality control of genotypes was performed based on MAF of higher than 0.01. This has resulted in obtaining a range of 19,876 to 19,956 polymorphic markers in generation 1 and 12,527–12,677 in generation 10 across different sub-scenarios. Subsequently, 10,000 markers from remaining informative markers in each generation were used for further analysis. To obtain means of allelic frequency, the simulated biallelic polymorphic genotypes were coded as 0, 1 and 2 for homozygote, heterozygote and homozygote respectively, and then divided by two. Mean allele frequencies for each SNP in each pool were calculated and arranged in a ‘design matrix’ with pool ID per family in columns and SNPs in rows, capturing the family variation.

### Statistical analysis

The matrix of mean family allelic frequency was used to construct the GRM (**G** matrix) and to compute the (working) inverse of the **G** matrix (G_*inv*_) as described by Ashraf et al. (2016). In contrast to their research, this study investigated scenarios where one, two and five pools were implemented as outlined above. To construct the **G** matrix; the matrix of allelic frequency estimates was first considered with SNPs in rows and samples in columns:

1 $${{{\mathbf{M}}}} = \left\{ {M_{ij}} \right\} = \left\{ {X_{ij} - \overline X _i} \right\}$$

where *X*_*ij*_ is the allele frequency at SNP *i* in family *j*, and **M** is the matrix of SNP allele frequencies with elements *M*_*ij*_ centred around the overall SNP *i* mean frequency, $\overline X _i$. Then, the GRM (**G**) was constructed:

2 $${{{\mathbf{G}}}} = \frac{{{{{\mathbf{M}}}}^\prime {{{\mathbf{M}}}}}}{K}$$

Here, *K* was estimated as ${\sum} {\overline X _i\left( {1 - \overline X _i} \right)}$. Since the **G** matrix was singular, it was inverted by the eigen-decomposition after adding a small constant to the zero eigenvalue:

3 $${{{\mathbf{G}}}}_{inv} = {{{\mathbf{E}}}}_v^\prime {{{\mathbf{\Lambda }}}}^{ - 1}{{{\mathbf{E}}}}_v$$

where **E**_*v*_ is the matrix of eigenvectors of **G** and **Λ** is the diagonal matrix of eigenvalues of **G**, modified as mentioned above.

Following this, the genetic parameters for all analyses were estimated using a REML method in ASReml-R package (Butler et al. 2009). The following model was fitted to the family pool data:

4 $${{{\mathbf{y}}}} = {\upmu}1 + {{{\mathbf{Zv}}}} + {{{\mathrm{e}}}}$$

where **y** is the vector of family phenotypic mean values, μ represents the constant term, **Z** defines the design matrix relating pools to families, **v** is the vector of random effects capturing the genetic value of the family, assumed ${{{\mathbf{v}}}}\sim N\left( {{{{\mathbf{0}}}},\sigma _a^2{{{\mathbf{G}}}}} \right)$, and **e** is the vector of residual errors, assumed ${{{\mathbf{e}}}}\sim N\left( {{{{\mathbf{0}}}},\sigma _e^2{{{\mathbf{I}}}}} \right)$, with $\sigma _a^2$ and $\sigma _e^2$ being the additive genetic and residual variances. The matrix **G** was provided as the GRM. Then, the heritability of family means (pools) $( {h_{pool}^2})$ in every scenario was estimated as $h_{pool}^2 = \frac{{\sigma _a^2}}{{\sigma _a^2 + \sigma _e^2}}$ using the REML estimates of the variance components.

The repeatability of family estimates was also estimated to investigate the accuracy of the family heritability estimates in S3 and S2 with 60-progeny for three heritability values, by first fitting a model to pool mean data similar to that in Eq. 4. However here, **v** is the vector of random family effects, assumed ${{{\mathbf{v}}}}\sim N\left( {{{{\mathbf{0}}}},\sigma _b^2{{{\mathbf{I}}}}} \right)$, and **e** is the vector of residual errors, assumed ${{{\mathbf{e}}}}\sim N\left( {{{{\mathbf{0}}}},\sigma _w^2{{{\mathbf{I}}}}} \right)$, with $\sigma _b^2$ and $\sigma _w^2$ being the between- and within-family variances. Subsequently, the repeatability was estimated as $\frac{{\sigma _b^2}}{{\sigma _b^2 + \sigma _w^2}}$ REML was also used to estimate the heritability from pedigree data for 100 and 200 family scenarios with 20, 40 and 60-progeny from S1 and S2 and, 50 and 400 families from S1, by first fitting the following animal model:

5 $${{{\mathbf{y}}}} = {\upmu}1 + {{{\mathbf{Zv}}}} + {{{\mathrm{e}}}}$$

where **y** is the vector of individual phenotypic values, μ represents the constant term, **Z** defines the design matrix relating records to appropriate random effects, **v** is the vector of animal random effects, assumed ${{{\mathbf{v}}}}\sim N\left( {{{{\mathbf{0}}}},\sigma _a^2A} \right)$, and **e** is the vector of residual errors, assumed ${{{\mathbf{e}}}}\sim N\left( {{{{\mathbf{0}}}},\sigma _e^2{{{\mathbf{I}}}}} \right)$, with $\sigma _a^2$ and $\sigma _e^2$ being the additive genetic and residual variances. The matrix **A** was provided as the NRM from the simulated pedigree. Next, the pedigree heritability was calculated as $h_{ped}^2 = \frac{{\sigma _a^2}}{{\sigma _a^2 + \sigma _e^2}}$ using the REML estimates of variance components.

The heritability of family means from S2 was compared with the family heritability ($h_f^2$) equation from Falconer and Mackay (1996):

6 $$h_f^2 = \frac{{1 + \left( {n - 1} \right)r}}{{1 + \left( {n - 1} \right)t}}h_{ped}^2$$

where $h_f^2$ is the family heritability, *n* is the number of progeny per family, *r* is the relationship between families being simply considered as 0.5 among full-sib family members e.g. in Falconer and Mackay (1996), and *t* is the intraclass correlation (ICC). The *t* value was obtained by fitting a model to individual animal data using a model of the form in Eq. 5, where **y** is the vector of individual phenotypic values, μ represents the constant term, **Z** defines the identity matrix (**I**) due to having one record per family, **v** is the vector of family random effects, assumed ${{{\mathbf{v}}}}\sim N\left( {{{{\mathbf{0}}}},\sigma _b^2{{{\mathbf{I}}}}} \right)$, and **e** is the vector of residual errors, assumed ${{{\mathbf{e}}}}\sim N\left( {{{{\mathbf{0}}}},\sigma _w^2{{{\mathbf{I}}}}} \right)$.

The heritability of family mean was averaged across replicates for presentation in graphs. The standard error of the estimates was obtained from their standard deviation:

$${{{\mathrm{SE = }}}}\frac{s}{{\sqrt n }}$$

where SE is standard error of heritability, *s* is equal to standard deviation of estimates across ten replicates, and *n* is the number of replicates.

All the analysis was conducted in R programming language (R Core Team 2020).

## Results

In this section, the results obtained from different factors affecting the estimation of heritability of family means are presented. Then, this is further expanded into the effect of the set (individual) trait heritability on estimated heritability of family means. Finally, the results of heritability of family means across different individual heritabilities are compared with corresponding estimates from Falconer’s family mean formula. Results are the average of estimates over ten replicates, whilst standard errors of sampling presented as error bars. However, in the supplementary file standard error associated with the individual estimates are provided.

### Parameters affecting the estimation of heritability of family means

The following sections summarise the effects of number of families, family size, number of pools and pooling size on heritability of family means. The first two sections present the results of single-pool based estimation of heritability of family means from S1 and S2 whilst the next two sections summarise the two- and five-pool results from S2 and the last section presents only the two-pool results from S3.

### Effect of the number of families on heritability of family means

**Fig. 1:**
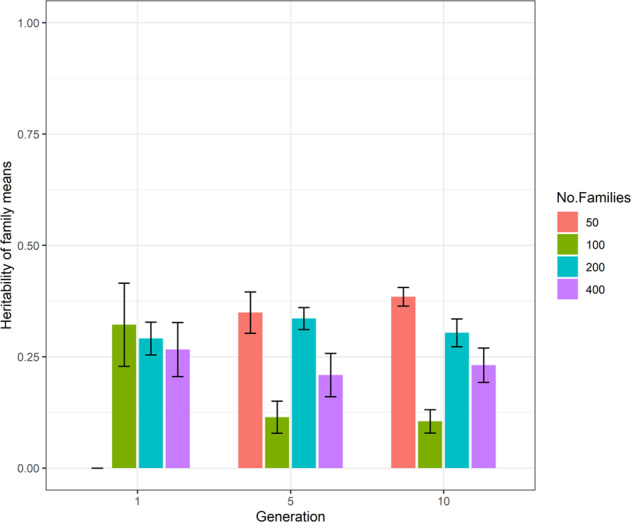
The estimates of heritability of family means of different number of families (scenario S1) based on single pool strategy in generations 1, 5 and 10. The results are averaged for ten replicates and standard errors of sampling shown as error bars. The *y*-axis represents the estimates of heritability of family means and the *x*-axis is the generation number. The coloured bar shows 50, 100, 200 and 400 number of families in ‘red brick’, ‘green’, ‘blue’ and ‘purple’ colour bars, respectively.

The estimates of heritability of family means for different numbers of families (50–400) with 20 progeny per family (scenario S1), based on a single pool per family are presented in Fig. 1. Detailed results of heritability of family mean are provided in Supplementary Table S1. The diagram demonstrates estimates in generations 1, 5 and 10. The figure shows that use of one pool resulted in unreliable estimation of heritability of family means, regardless of the number of families. This was indicated by the high variation of results in all families across generations. In addition, detailed results of heritability of family means presented in Supplementary Table S1 shows that estimates across generations and replications for all family numbers were not repeatable. Furthermore, standard error of heritability of family means estimates in the Supplementary Table S1 were high, rendering the results for single pools unreliable.

### Effect of the size of families on heritability of family means

**Fig. 2:**
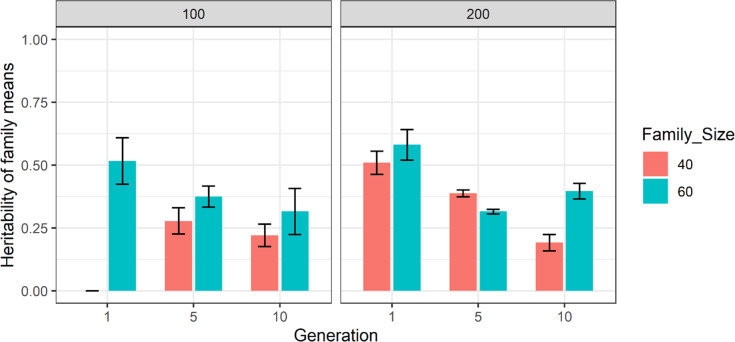
The estimates of heritability of family means for 100 and 200 families in conjunction with 40 and 60 progeny per family (scenario S2) using a single pool scheme in generations 1, 5 and 10. The *y*-axis is the estimate of heritability of family means and the *x*-axis is the generation number. The legend bar shows family sizes of 40 and 60 in ‘red brick’ and ‘green’ respectively. The results are averages over ten replicates and error bars represent the standard error of samplings over ten replicates.

The heritability of family mean estimates over family sizes of 40 and 60 (scenario S2) in the 100- and 200-family scenarios is depicted in Fig. 2. The estimates are provided for generations 1, 5 and 10 and are averaged over ten independent replicates using a single pool. The results showed that increasing the size of families in the 100- or 200-family scenarios did not estimate the heritability of family means reliably whenever one pool is considered. This is reflected by the large standard errors of samplings, resulting in the unrepeatable patterns of changing the estimates over replicates and generations as also demonstrated in Supplementary Table S1.

### Effect of number of pools on heritability of family means

**Fig. 3:**
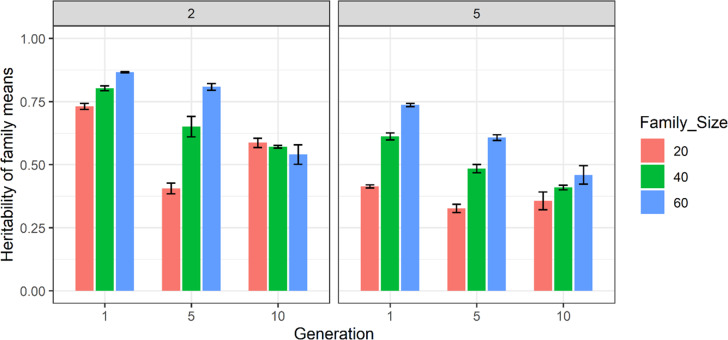
The estimates of heritability of family means using two and five pools of 100 families from S1 and S2 scenarios in generations 1, 5 and 10. The *y*-axis is the estimate of heritability of family means and the *x*-axis is generation number. The legend colour bar shows family sizes of 20, 40 and 60 in ‘red brick’, ‘green’ and ‘blue’ respectively. Results are averaged over ten replicates and standard error of sampling are presented as error bars.

The effect of using two and five pools in estimating heritability of family means with 100 families from S1 and S2 scenarios is shown in Fig. 3. Using both two and five pools, heritability estimates decreased from generation 1 to 10. However, this was not true of the 20-progeny per family scenario from generation 5–10. The detailed results of heritability of family means are provided in Supplementary Table S2. Overall, the 20-progeny scenario estimates did not show consistency over replicates. The standard errors of estimates were generally high but much smaller as compared to single pool, and the extent of change was not consistent across replicates. Hence, estimates of heritability of family means were not estimated with high precision using 100 families for S1 and S2 scenarios using two and five pools.

**Fig. 4:**
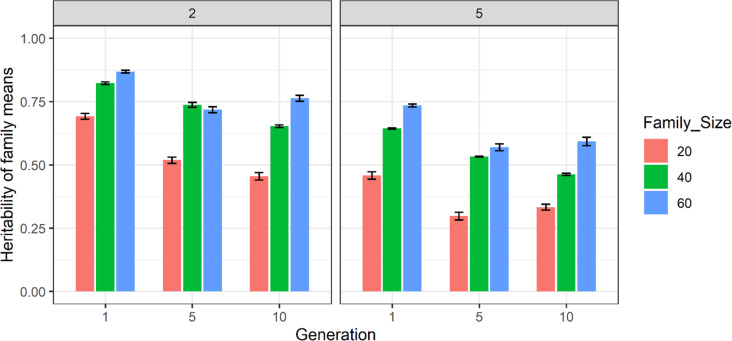
The estimates of heritability of family means using two and five pools of 200 families from S1 and S2 scenarios in generations 1, 5 and 10. The *y*-axis is the estimate of heritability of family means and the *x*-axis is generation number. The legend colour bar shows family sizes 20, 40 and 60 in ‘red brick’, ‘green’ and ‘blue’ respectively. Results are averaged over ten replicates and standard error of sampling are presented as error bars.

In the 200-families scenario from S1 and S2 which is depicted in Fig. 4 and Supplementary Table S2, the family setting showed more consistent estimates and less fluctuation between replicates in comparison to the 100-families scheme as presented using error bars. However, the estimate of heritability of family means using 20 progeny per family was less consistent across replicates in both two- and five-pool strategies compared to 40 and 60 progeny per family. Overall, 60 progeny per family using two pools showed more consistency in pattern across replicates. Consequently, this scenario has been chosen for further analysis.

### Effect of the number of pools on the genetic relationship matrix

**Fig. 5:**
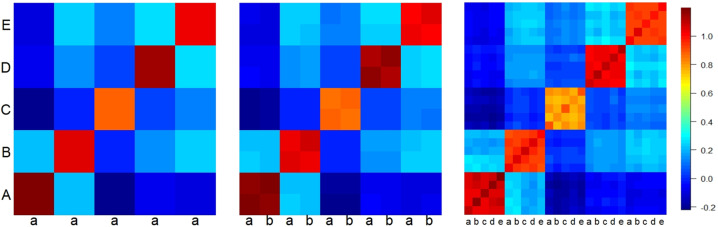
The effect of genotype pooling on genetic relationship matrix shown for the first five of 200 families and 60 progeny per family simulation in generation 5 of replicate 1. The diagonal blocks show within-pool genetic relationships and off-diagonal between pools. A: family 1, B: family 2, C: family 3, D: family 4 and E: family 5. a: 1st pool, b: 2nd pool, c: 3rd pool, d: 4th pool and e: 5th pool in each family. One pool has generated a 5 × 5 matrix, two pools a 10 × 10 matrix and five pools a 25 × 25 matrix.

The effect of pooling the genotypes on within- and between-family relationships for scenario S2 is presented in Fig. 5, showing only the first five out of 200 families from a 60-progeny per family scenario for the purpose of illustration. Capital and small letters are presenting different families and pools respectively. Using this structure, from the one, two and five-pool schemes, there were 60, 30 and 12 progeny per pool, respectively. The graph indicates that the application of two and five pools has generated not only a larger number of between-family relationships but also within-family relationships, e.g., five pools have generated a 5 × 5 GRM within each family. Although within-family relationships were high, the relationships across families changed from small to high across different generations. For example, the extent of between-family relationships for the sub-scenario used in this section was ranging from −0.091 to 0.216 in G01, −0.742 to 0.928 in G05 and −0.579 to 0.848 in G10.

### Effect of pool size on heritability of family means

**Fig. 6:**
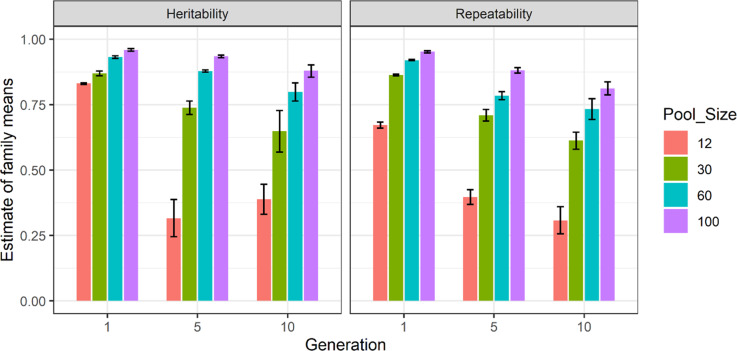
The heritability of family means and repeatability estimates using two pools for 50 families and 200 progeny per family and a pool size of 12, 30, 60 and 100 in generations 1, 5 and 10. The *y*-axis is the heritability of family means and repeatability and, the *x*-axis is the generation number. The legend bar shows pool sizes of 12, 30, 60 and 100 in ‘red brick’, ‘green’, ‘blue’ and ‘purple’ respectively.

Estimates of family mean heritability for pool sizes of 12, 30, 60 and 100 for 50 families of 200 progeny each from scenario S3, along with their associated repeatability values are presented in Fig. 6. The results are provided for the two-pool scenario using trait heritability of 0.3. The detailed results of heritability of family means and their associated repeatabilities for ten replicates are provided in Supplementary Table S3. Standard errors of sampling are shown as error bars in the figure whilst standard errors of all estimates are provided in the supplementary table. Using 12 progeny per pool in each family, large standard errors and inconsistent trends were obtained whilst this was not the case using a higher number of individuals per pool. In addition, increasing the number of progeny per pool increased the heritability estimates of family means towards one. However, the standard errors were high even using 100 progeny per pool per family. This can be due to the small number of families used in this experiment.

### Effect of trait heritability on estimate of heritability of family means

**Fig. 7:**
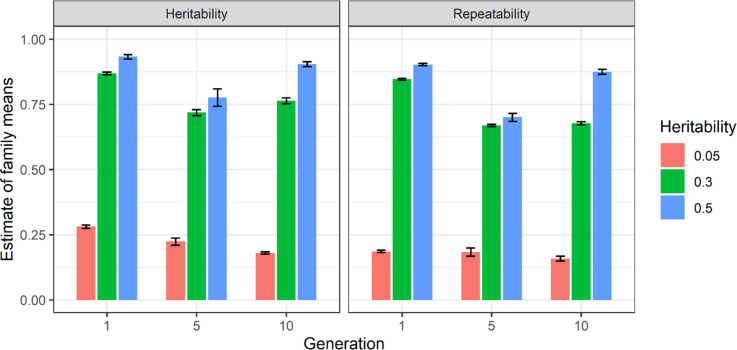
The estimates of heritability and repeatability of family means using two pools of 200-family and 60 progeny per family scenario in generations 1, 5 and 10. The *y*-axis is the heritability of family means estimate and repeatability and the *x*-axis is generation number. The legend bar shows trait heritabilities of 0.05, 0.3 and 0.5 in ‘red brick’, ‘green’ and ‘blue’ respectively. Standard error of samplings shown represented as error bars.

The estimates of heritability of family means for the trait heritabilities of 0.05, 0.3 and 0.5 implemented in G0 and their associated repeatabilities are provided in Fig. 7 for ten replicates in G01, G05 and G10. The diagram is presented for the scenario with 200 families of 60 progeny/family from S2 scenario split into two pools. The detailed results of heritability of family means and repeatabilities are provided in Supplementary Table S2. Using trait heritabilities of 0.05, 0.3 and 0.5, estimates of heritability of family means across replicates were consistent as shown by error bars. Using all trait heritabilities, the heritability of family means was always greater than their respective repeatability values.

### Heritability of family means vs Falconer’s family heritability estimates

The pedigree heritabilities ($h_{ped}^2$), ICCs, family heritabilities inferred from Falconer’s formula ($h_f^2$) and heritability of family means ($h_{pool}^2$) with their standards error of sampling in the parentheses are provided in Table 2. The results are presented for G01, G05 and G10 averaged over ten independent replicates. Estimated individual heritabilities were in expected range in generations based on input heritabilities of the trait and selection method. The intraclass correlations, on the other hand, were very small resulting in infeasible values calculated with the Falconer formula, including most of the values for moderate and high trait heritabilities returning values greater than one.

**Table 2:** Estimate of pedigree and family heritability, and intraclass correlation for different initial trait heritability values in which are averaged over ten replicates for three generations (generation 1, 5 and 10) using 200 families of 60 progeny per family.

	*h²*: 0.05				*h²*: 0.3				*h²*: 0.5			
Generation	$h_{ped}^2$	*ICC*	$h_f^2$	$h_{pool}^2$	$h_{ped}^2$	*ICC*	$h_f^2$	$h_{pool}^2$		*ICC*	$h_f^2$	$h_{pool}^2$
1	0.054 (0.017)	0.031 (0.008)	0.446 (0.121)	0.281 (0.021)	0.314 (0.018)	0.159 (0.018)	0.933 (0.097)	0.875 (0.082)	0.489 (0.018)	0.228 (0.017)	1.003 (0.080)	0.932 (0.027)
5	0.041 (0.007)	0.016 (0.004)	0.515 (0.221)	0.224 (0.045)	0.230 (0.015)	0.066 (0.005)	1.113 (0.073)	1.113 (0.079)	0.482 (0.018)	0.123 (0.012)	1.664 (0.144)	0.776 (0.106)
10	0.055 (0.005)	0.015 (0.002)	0.626 (0.094)	0.181 (0.013)	0.226 (0.008)	0.061 (0.004)	1.280 (0.030)	1.278 (0.030)	0.493 (0.008)	0.104 (0.005)	1.909 (0.074)	0.904 (0.030)

*$h_{ped}^2$ the pedigree-based heritability, *ICC* the intraclass correlation, $h_f^2$ family heritability resulted from Falconer formula, $h_{pool}^2$ heritability of family means and numbers in parenthesis are standard errors of samplings.

*h*²: 0.05, 0.3 and 0.5 are the initial trait heritabilities implemented in the base generation (G0) in QMSim.

## Discussion

This study illustrated the application of family-pooled phenotypic and genotypic data for estimating the heritability of family means. The effect of different parameters including the number of families, size of family, number and size of pools and individual heritability was explored. Finally, the estimates of heritability of family means using pooled phenotypic and genotypic data, together with pedigree information, were compared with family heritability estimates obtained using Falconer’s formula, where the family heritability is explained by the relationship between family members, intraclass correlation of within-family phenotypic values and individual heritability.

The results of this simulation study showed that using a single pool did not result in accurate estimation of heritability of family means, even after either increasing the number of families (Fig. 1) or size of families (Fig. 2). The inaccuracy in estimating the heritability of family means was reflected in high standard errors and inconsistent patterns of estimates across replicates. In contrast to a single pool, using two and five pools (Figs. 3 and 4) resulted in higher and more accurate estimates especially using the 200-family scenario described under S2. This can be seen in Fig. 5 where application of two and five pools per family has generated both within- and between-family relationships. Although it was not investigated in this study, Ashraf et al. (2016) highlighted that capturing within- and between-family relationships accompanied by obtaining the non-additive and genotype by environmental (G × E) component can result in more accurate estimates of heritability of family means using a pooling strategy.

As compared to the five-pool strategy, higher and more precise estimates of heritability of family means were obtained using the two-pool scenario. This can be explained by reduction of individual residual variance ($\sigma _e^2$). Let us assume that *n* is the pool size and family size is constant, e.g., 60. When dividing the family in two pools, the value of *n* would be 30 whilst for five-pool scenario that would be 12. Consequently, an *n* of 30 can lower the residual variance ($\sigma _e^2$) compared to a pool of sample size 12, considering that approximately, $\sigma _e^2 = \frac{{\sigma _i^2}}{n}$, where $\sigma _i^2$ is the residual variance at the individual level. In this study, using five as compared to two pools resulted in smaller *n* from the same family size and consequently lower estimates for heritability of family means.

Among the scenarios with two pools, using 200 families of 60 progeny showed greater consistency across generations and replicates. Although 40 progeny using the same number of families was also consistent, considering $\sigma _e^2 = \frac{{\sigma _i^2}}{n}$, one would expect to obtain lower estimates using 40 progeny. However, using 40 progeny resulted in higher heritability estimates compared to 60 progeny in generation 5 (Fig. 4). Ashraf et al. (2016) also presented results on family heritability using pooled phenotypic and genotypic data. They used a maximum of three individuals per family, two pools per family and 990 families, whilst in our study the number of individuals per pool was 30. As the individual heritability is not reported in their study, it is difficult to make further comparisons.

As shown in Fig. 6, when the number of progeny per pool was increased, the estimate of heritability of family means approached unity, this occurs because the residual variance approaches zero and hence the heritability approaches one. This change in estimates is supported by e.g. Falconer and Mackay (1996), who mentioned that the larger the family size, the closer the phenotypic value of family mean to its genotypic value, i.e. its true breeding value.

While Falconer’s formula was able to explain why increasing pool size increased the heritability of family means, its estimates of family mean heritability were out of bounds as shown in Table 2. The inability of Falconer’s formula to estimate the family mean heritability in this experiment was due to a much lower ICC as compared to coefficient of relationship (the coefficient of relationship in this study for full-sib families were considered as 0.5 as previously stated e.g. in Falconer and Mackay (1996)). Consequently, lower *t* (with a small standard error) values as compared to coefficient of relationship has resulted in Falconer’s equation to estimate the family heritability inaccurately.

The estimates of heritability of family means and repeatability in Figs. 6 and 7 showed that heritability of family mean estimates were usually higher than repeatability measurements. These results contradict Falconer and Mackay (1996) which mentions that repeatability sets the upper limit of heritability. It should be noted that Falconer and Mackay (1996) rule applies to heritability and repeatability at individual/population level not on pools. In general, two main condition should be fulfilled to have higher repeatability than heritability, namely, high residual (environmental) variation and substantial non-additive genetic variance (Boake 1989). The residual variation using pooled-family data in this study was low (Supplementary Table 2). In addition, both heritability of family means, and repeatability were high as presented in Figs. 6 and 7 which according to Boake (1989) should be the result of small environmental variation and additive nature of genetic variance. Therefore, two criteria for obtaining higher repeatability measurements compared to heritability were not met here. Furthermore, genotypic data was used to estimate heritability of family means in this study whilst repeatability was obtained using pedigree. Therefore, using different source of data may also be contributed to estimations of repeatability and heritability of family means.

Overall, this study demonstrated that using pooled phenotypic and genotypic data can be effectively used in constructing the GRM and estimating the heritability of family means. However, this requires having access to data from a large number of families, e.g., 200, with at least 60 progeny per family within the boundary of parameters tested in this study. Nevertheless, this can be further investigated using a greater number of families, family sizes, number of pools and varying pool size. In addition, simulation studies may not necessarily mirror the specific situations on commercial farms. For example, whilst in this study we assumed that families are kept separately at least before measuring their phenotypic values and genotyping, in commercial aquaculture settings, families are kept in a large cohort of communal progeny-rearing environment. Hence, parentage assignment may be required to ensure that every family has equal contribution to sampling. This might be problematic where the genotyping budget is restricted. In addition, if more families and progeny per family are available in commercial farming, even a strategy without pooling might outperform pooling scenarios as stated by Technow and Totir (2015). Therefore, it is essential to extend the approach used in this study and explore it against data and constraints imposed from commercial breeding farms. Furthermore, the focus on the current study was on estimation of heritability based on pooled genotypes and quantitative phenotypes. The approach can be extended to the survival traits in future studies. Future studies should also estimate the accuracy of prediction and genetic gain for black tiger prawn as it has been investigated for other species in several other studies e.g., Bell et al. (2017), Alexandre et al. (2019), Alexandre et al. (2020) and Baller et al. (2020), to compare the result of within-family pooling with population-wise data.

## Supplementary information

S1_Heritability of family means (pool) and pedigree (ped) Heritability, their associated additive (σ_a_²), residual (σ_e_²) variance and respective standard erros (SE).

S2_Heritability of family means (pool) and pedigree (ped) Heritability, their associated additive (σ_a_²), residual (σ_e_²) variance and respective standard erros (SE).

S3_Heritability of family means (pool), repeatability (t) and their respective standard erros (SE). The results are for two-pool Scenario from S3 for trait heritability of 0.3.
