# Supplementary material for: Estimating heritability using family-pooled phenotypic and genotypic data: a simulation study applied to aquaculture
Source: Heredity (Edinb). 2022 Jan 31;128(3):178–86. doi: 10.1038/s41437-022-00502-8 (PMC8897491; doi:10.1038/s41437-022-00502-8)
Supplement: Supplementary file 1 — S1_Heritability of family means (pool) and pedigree (ped) Heritability, their associated additive (σa²), residual (σe²) variance and respective standard erros (SE). [file 41437_2022_502_MOESM1_ESM.pdf]

**Supplementary Table S1. Heritability of family means (pool) and pedigree (ped) Heritability, their associated additive ($\sigma_a^2$), residual ($\sigma_e^2$) variance and respective standard erros (SE). The results are for single pool Scenario and for trait heritability of 0.3.**

| Generation | Replicate | No. Families | Family Size | $h^2$ (pool) | SE | $\sigma_a^2$ (pool) | SE | $\sigma_e^2$ (pool) | SE | $h^2$ (ped) | SE | $\sigma_a^2$ (ped) | SE | $\sigma_e^2$ (ped) | SE |
|---|---|---|---|---|---|---|---|---|---|---|---|---|---|---|---|
| 1 | 1 | 50 | 20 | 0.000 | 0.000 | 0.000 | 0.000 | 0.334 | 0.010 | 0.375 | 0.068 | 0.402 | 0.083 | 0.671 | 0.055 |
| 1 | 2 | 50 | 20 | 0.000 | 0.000 | 0.000 | 0.000 | 0.107 | 0.135 | 0.211 | 0.055 | 0.213 | 0.061 | 0.795 | 0.050 |
| 1 | 3 | 50 | 20 | 0.000 | 0.000 | 0.000 | 0.000 | 0.101 | 0.152 | 0.247 | 0.056 | 0.231 | 0.059 | 0.705 | 0.047 |
| 1 | 4 | 50 | 20 | 0.000 | 0.000 | 0.000 | 0.000 | 0.344 | 0.010 | 0.264 | 0.062 | 0.280 | 0.079 | 0.780 | 0.054 |
| 1 | 5 | 50 | 20 | 0.000 | 0.000 | 0.000 | 0.000 | 0.106 | 0.138 | 0.247 | 0.056 | 0.248 | 0.067 | 0.755 | 0.043 |
| 1 | 6 | 50 | 20 | 0.000 | 0.000 | 0.000 | 0.000 | 0.160 | 0.176 | 0.167 | 0.056 | 0.158 | 0.059 | 0.787 | 0.049 |
| 1 | 7 | 50 | 20 | 0.000 | 0.000 | 0.000 | 0.000 | 0.289 | 0.004 | 0.286 | 0.055 | 0.315 | 0.057 | 0.785 | 0.051 |
| 1 | 8 | 50 | 20 | 0.000 | 0.000 | 0.000 | 0.000 | 0.062 | 0.156 | 0.278 | 0.056 | 0.261 | 0.069 | 0.678 | 0.055 |
| 1 | 9 | 50 | 20 | 0.000 | 0.000 | 0.000 | 0.000 | 0.172 | 0.166 | 0.299 | 0.066 | 0.335 | 0.079 | 0.785 | 0.045 |
| 1 | 10 | 50 | 20 | 0.000 | 0.000 | 0.000 | 0.000 | 0.166 | 0.156 | 0.283 | 0.056 | 0.297 | 0.063 | 0.753 | 0.050 |
| 5 | 1 | 50 | 20 | 0.288 | 0.161 | 0.030 | 0.013 | 0.073 | 0.024 | 0.325 | 0.074 | 0.385 | 0.094 | 0.801 | 0.061 |
| 5 | 2 | 50 | 20 | 0.382 | 0.227 | 0.069 | 0.049 | 0.111 | 0.037 | 0.271 | 0.064 | 0.269 | 0.073 | 0.723 | 0.052 |
| 5 | 3 | 50 | 20 | 0.331 | 0.202 | 0.026 | 0.017 | 0.053 | 0.026 | 0.146 | 0.051 | 0.131 | 0.048 | 0.769 | 0.043 |
| 5 | 4 | 50 | 20 | 0.594 | 0.207 | 0.443 | 0.017 | 0.303 | 0.021 | 0.296 | 0.070 | 0.331 | 0.094 | 0.788 | 0.059 |
| 5 | 5 | 50 | 20 | 0.151 | 0.200 | 0.006 | 0.062 | 0.035 | 0.030 | 0.283 | 0.065 | 0.293 | 0.075 | 0.742 | 0.052 |
| 5 | 6 | 50 | 20 | 0.559 | 0.153 | 0.181 | 0.013 | 0.143 | 0.025 | 0.277 | 0.054 | 0.310 | 0.044 | 0.811 | 0.045 |
| 5 | 7 | 50 | 20 | 0.155 | 0.183 | 0.006 | 0.017 | 0.034 | 0.023 | 0.300 | 0.068 | 0.337 | 0.021 | 0.785 | 0.036 |
| 5 | 8 | 50 | 20 | 0.365 | 0.187 | 0.063 | 0.051 | 0.109 | 0.036 | 0.262 | 0.069 | 0.274 | 0.100 | 0.771 | 0.053 |
| 5 | 9 | 50 | 20 | 0.394 | 0.156 | 0.083 | 0.017 | 0.128 | 0.029 | 0.264 | 0.060 | 0.274 | 0.074 | 0.764 | 0.051 |
| 5 | 10 | 50 | 20 | 0.270 | 0.171 | 0.028 | 0.030 | 0.075 | 0.019 | 0.248 | 0.075 | 0.267 | 0.035 | 0.811 | 0.040 |
| 10 | 1 | 50 | 20 | 0.460 | 0.197 | 0.089 | 0.022 | 0.105 | 0.027 | 0.249 | 0.067 | 0.246 | 0.074 | 0.741 | 0.050 |
| 10 | 2 | 50 | 20 | 0.270 | 0.174 | 0.072 | 0.026 | 0.195 | 0.029 | 0.259 | 0.067 | 0.232 | 0.070 | 0.663 | 0.046 |
| 10 | 3 | 50 | 20 | 0.366 | 0.175 | 0.137 | 0.021 | 0.237 | 0.029 | 0.455 | 0.083 | 0.510 | 0.109 | 0.611 | 0.059 |
| 10 | 4 | 50 | 20 | 0.414 | 0.161 | 0.080 | 0.025 | 0.113 | 0.032 | 0.348 | 0.051 | 0.354 | 0.072 | 0.662 | 0.040 |
| 10 | 5 | 50 | 20 | 0.391 | 0.154 | 0.091 | 0.015 | 0.142 | 0.031 | 0.331 | 0.057 | 0.339 | 0.083 | 0.686 | 0.045 |
| 10 | 6 | 50 | 20 | 0.422 | 0.202 | 0.198 | 0.027 | 0.271 | 0.033 | 0.366 | 0.063 | 0.449 | 0.080 | 0.779 | 0.048 |
| 10 | 7 | 50 | 20 | 0.276 | 0.167 | 0.097 | 0.014 | 0.255 | 0.036 | 0.301 | 0.063 | 0.327 | 0.091 | 0.760 | 0.050 |
| 10 | 8 | 50 | 20 | 0.446 | 0.178 | 0.092 | 0.022 | 0.114 | 0.033 | 0.297 | 0.069 | 0.327 | 0.088 | 0.773 | 0.057 |
| 10 | 9 | 50 | 20 | 0.430 | 0.185 | 0.096 | 0.017 | 0.127 | 0.037 | 0.397 | 0.071 | 0.448 | 0.099 | 0.681 | 0.060 |
| 10 | 10 | 50 | 20 | 0.371 | 0.159 | 0.069 | 0.024 | 0.117 | 0.037 | 0.296 | 0.077 | 0.275 | 0.103 | 0.653 | 0.065 |
| 1 | 1 | 100 | 20 | 0.000 | 0.000 | 0.000 | 0.000 | 0.205 | 0.029 | 0.313 | 0.049 | 0.323 | 0.058 | 0.706 | 0.041 |
| 1 | 2 | 100 | 20 | 0.556 | 0.572 | 0.154 | 0.103 | 0.123 | 0.102 | 0.280 | 0.046 | 0.286 | 0.053 | 0.734 | 0.040 |
| 1 | 3 | 100 | 20 | 0.216 | 0.592 | 0.036 | 0.099 | 0.131 | 0.105 | 0.273 | 0.045 | 0.258 | 0.048 | 0.687 | 0.037 |
| 1 | 4 | 100 | 20 | 0.000 | 0.000 | 0.000 | 0.000 | 0.213 | 0.029 | 0.293 | 0.046 | 0.303 | 0.056 | 0.732 | 0.042 |
| 1 | 5 | 100 | 20 | 0.871 | 0.563 | 0.155 | 0.103 | 0.023 | 0.102 | 0.271 | 0.045 | 0.273 | 0.052 | 0.736 | 0.035 |
| 1 | 6 | 100 | 20 | 0.246 | 0.596 | 0.046 | 0.101 | 0.141 | 0.115 | 0.241 | 0.043 | 0.236 | 0.046 | 0.741 | 0.038 |
| 1 | 7 | 100 | 20 | 0.000 | 0.000 | 0.000 | 0.000 | 0.216 | 0.031 | 0.281 | 0.046 | 0.291 | 0.051 | 0.744 | 0.039 |
| 1 | 8 | 100 | 20 | 0.556 | 0.565 | 0.153 | 0.103 | 0.122 | 0.102 | 0.286 | 0.045 | 0.284 | 0.053 | 0.710 | 0.041 |
| 1 | 9 | 100 | 20 | 0.520 | 0.543 | 0.155 | 0.104 | 0.143 | 0.116 | 0.293 | 0.048 | 0.310 | 0.056 | 0.749 | 0.036 |
| 1 | 10 | 100 | 20 | 0.253 | 0.586 | 0.047 | 0.105 | 0.139 | 0.105 | 0.287 | 0.045 | 0.294 | 0.050 | 0.731 | 0.040 |

| | | | | | | | | | | | | | | | |
|---|---|---|---|---|---|---|---|---|---|---|---|---|---|---|---|
| 5 | 1 | 100 | 20 | 0.000 | 0.000 | 0.000 | 0.000 | 0.133 | 0.019 | 0.263 | 0.052 | 0.275 | 0.062 | 0.769 | 0.043 |
| 5 | 2 | 100 | 20 | 0.238 | 0.225 | 0.056 | 0.035 | 0.179 | 0.027 | 0.252 | 0.049 | 0.243 | 0.054 | 0.721 | 0.039 |
| 5 | 3 | 100 | 20 | 0.056 | 0.171 | 0.011 | 0.017 | 0.185 | 0.020 | 0.156 | 0.040 | 0.141 | 0.038 | 0.765 | 0.034 |
| 5 | 4 | 100 | 20 | 0.000 | 0.000 | 0.000 | 0.000 | 0.132 | 0.018 | 0.258 | 0.050 | 0.264 | 0.062 | 0.761 | 0.042 |
| 5 | 5 | 100 | 20 | 0.249 | 0.215 | 0.060 | 0.038 | 0.181 | 0.022 | 0.228 | 0.047 | 0.217 | 0.055 | 0.733 | 0.039 |
| 5 | 6 | 100 | 20 | 0.061 | 0.178 | 0.012 | 0.017 | 0.184 | 0.021 | 0.246 | 0.042 | 0.250 | 0.037 | 0.763 | 0.035 |
| 5 | 7 | 100 | 20 | 0.000 | 0.000 | 0.000 | 0.000 | 0.182 | 0.019 | 0.266 | 0.051 | 0.272 | 0.028 | 0.750 | 0.031 |
| 5 | 8 | 100 | 20 | 0.310 | 0.235 | 0.058 | 0.034 | 0.129 | 0.025 | 0.242 | 0.049 | 0.238 | 0.064 | 0.747 | 0.039 |
| 5 | 9 | 100 | 20 | 0.102 | 0.171 | 0.021 | 0.017 | 0.184 | 0.023 | 0.238 | 0.045 | 0.235 | 0.052 | 0.753 | 0.038 |
| 5 | 10 | 100 | 20 | 0.126 | 0.170 | 0.019 | 0.022 | 0.132 | 0.017 | 0.237 | 0.052 | 0.238 | 0.035 | 0.766 | 0.033 |
| 10 | 1 | 100 | 20 | 0.124 | 0.155 | 0.014 | 0.018 | 0.099 | 0.021 | 0.225 | 0.049 | 0.217 | 0.053 | 0.748 | 0.038 |
| 10 | 2 | 100 | 20 | 0.185 | 0.177 | 0.022 | 0.022 | 0.097 | 0.023 | 0.253 | 0.051 | 0.240 | 0.055 | 0.710 | 0.037 |
| 10 | 3 | 100 | 20 | 0.000 | 0.000 | 0.000 | 0.000 | 0.151 | 0.022 | 0.352 | 0.058 | 0.356 | 0.071 | 0.655 | 0.042 |
| 10 | 4 | 100 | 20 | 0.156 | 0.150 | 0.017 | 0.019 | 0.092 | 0.023 | 0.294 | 0.043 | 0.289 | 0.056 | 0.695 | 0.033 |
| 10 | 5 | 100 | 20 | 0.197 | 0.175 | 0.024 | 0.018 | 0.098 | 0.024 | 0.284 | 0.045 | 0.285 | 0.058 | 0.718 | 0.035 |
| 10 | 6 | 100 | 20 | 0.079 | 0.180 | 0.015 | 0.022 | 0.174 | 0.024 | 0.303 | 0.047 | 0.327 | 0.060 | 0.754 | 0.037 |
| 10 | 7 | 100 | 20 | 0.000 | 0.000 | 0.000 | 0.000 | 0.170 | 0.025 | 0.256 | 0.049 | 0.254 | 0.062 | 0.738 | 0.039 |
| 10 | 8 | 100 | 20 | 0.203 | 0.167 | 0.025 | 0.020 | 0.098 | 0.025 | 0.282 | 0.051 | 0.289 | 0.064 | 0.737 | 0.041 |
| 10 | 9 | 100 | 20 | 0.000 | 0.000 | 0.000 | 0.000 | 0.095 | 0.026 | 0.310 | 0.053 | 0.323 | 0.066 | 0.721 | 0.043 |
| 10 | 10 | 100 | 20 | 0.104 | 0.160 | 0.011 | 0.019 | 0.095 | 0.026 | 0.261 | 0.055 | 0.246 | 0.068 | 0.695 | 0.045 |
| 1 | 1 | 100 | 40 | 0.000 | 0.000 | 0.000 | 0.000 | 0.152 | 0.022 | 0.263 | 0.038 | 0.261 | 0.043 | 0.731 | 0.029 |
| 1 | 2 | 100 | 40 | 0.000 | 0.000 | 0.000 | 0.000 | 0.151 | 0.022 | 0.281 | 0.040 | 0.263 | 0.043 | 0.673 | 0.029 |
| 1 | 3 | 100 | 40 | 0.000 | 0.000 | 0.000 | 0.000 | 0.182 | 0.026 | 0.316 | 0.043 | 0.321 | 0.052 | 0.693 | 0.033 |
| 1 | 4 | 100 | 40 | 0.000 | 0.000 | 0.000 | 0.000 | 0.171 | 0.022 | 0.277 | 0.038 | 0.279 | 0.043 | 0.730 | 0.029 |
| 1 | 5 | 100 | 40 | 0.000 | 0.000 | 0.000 | 0.000 | 0.151 | 0.026 | 0.296 | 0.040 | 0.291 | 0.052 | 0.693 | 0.033 |
| 1 | 6 | 100 | 40 | 0.000 | 0.000 | 0.000 | 0.000 | 0.171 | 0.022 | 0.300 | 0.038 | 0.313 | 0.043 | 0.729 | 0.029 |
| 1 | 7 | 100 | 40 | 0.000 | 0.000 | 0.000 | 0.000 | 0.165 | 0.022 | 0.290 | 0.040 | 0.291 | 0.043 | 0.713 | 0.029 |
| 1 | 8 | 100 | 40 | 0.000 | 0.000 | 0.000 | 0.000 | 0.199 | 0.026 | 0.283 | 0.043 | 0.278 | 0.052 | 0.703 | 0.033 |
| 1 | 9 | 100 | 40 | 0.000 | 0.000 | 0.000 | 0.000 | 0.165 | 0.022 | 0.296 | 0.038 | 0.309 | 0.043 | 0.734 | 0.029 |
| 1 | 10 | 100 | 40 | 0.000 | 0.000 | 0.000 | 0.000 | 0.170 | 0.026 | 0.307 | 0.040 | 0.301 | 0.052 | 0.679 | 0.033 |
| 5 | 1 | 100 | 40 | 0.574 | 0.193 | 0.051 | 0.023 | 0.038 | 0.015 | 0.202 | 0.036 | 0.188 | 0.037 | 0.741 | 0.026 |
| 5 | 2 | 100 | 40 | 0.380 | 0.181 | 0.046 | 0.026 | 0.075 | 0.020 | 0.300 | 0.045 | 0.308 | 0.055 | 0.720 | 0.032 |
| 5 | 3 | 100 | 40 | 0.092 | 0.135 | 0.006 | 0.009 | 0.059 | 0.011 | 0.162 | 0.032 | 0.143 | 0.030 | 0.737 | 0.023 |
| 5 | 4 | 100 | 40 | 0.197 | 0.181 | 0.016 | 0.023 | 0.064 | 0.015 | 0.196 | 0.036 | 0.178 | 0.037 | 0.730 | 0.026 |
| 5 | 5 | 100 | 40 | 0.279 | 0.135 | 0.020 | 0.009 | 0.052 | 0.020 | 0.234 | 0.032 | 0.228 | 0.030 | 0.746 | 0.023 |
| 5 | 6 | 100 | 40 | 0.233 | 0.193 | 0.016 | 0.023 | 0.053 | 0.015 | 0.199 | 0.036 | 0.182 | 0.037 | 0.732 | 0.026 |
| 5 | 7 | 100 | 40 | 0.383 | 0.181 | 0.031 | 0.026 | 0.051 | 0.020 | 0.195 | 0.045 | 0.177 | 0.055 | 0.729 | 0.032 |
| 5 | 8 | 100 | 40 | 0.210 | 0.135 | 0.023 | 0.009 | 0.088 | 0.011 | 0.223 | 0.032 | 0.210 | 0.030 | 0.732 | 0.023 |
| 5 | 9 | 100 | 40 | 0.020 | 0.181 | 0.001 | 0.023 | 0.041 | 0.015 | 0.245 | 0.036 | 0.232 | 0.037 | 0.715 | 0.026 |
| 5 | 10 | 100 | 40 | 0.412 | 0.135 | 0.039 | 0.009 | 0.056 | 0.020 | 0.227 | 0.032 | 0.217 | 0.030 | 0.738 | 0.023 |
| 10 | 1 | 100 | 40 | 0.044 | 0.119 | 0.003 | 0.008 | 0.063 | 0.012 | 0.201 | 0.037 | 0.185 | 0.039 | 0.735 | 0.024 |
| 10 | 2 | 100 | 40 | 0.281 | 0.188 | 0.018 | 0.014 | 0.047 | 0.011 | 0.158 | 0.032 | 0.147 | 0.033 | 0.781 | 0.023 |
| 10 | 3 | 100 | 40 | 0.301 | 0.159 | 0.024 | 0.014 | 0.055 | 0.013 | 0.205 | 0.037 | 0.184 | 0.037 | 0.714 | 0.024 |
| 10 | 4 | 100 | 40 | 0.094 | 0.119 | 0.008 | 0.008 | 0.078 | 0.011 | 0.138 | 0.037 | 0.128 | 0.039 | 0.798 | 0.024 |
| 10 | 5 | 100 | 40 | 0.142 | 0.159 | 0.009 | 0.014 | 0.052 | 0.013 | 0.185 | 0.037 | 0.165 | 0.037 | 0.730 | 0.023 |
| 10 | 6 | 100 | 40 | 0.085 | 0.119 | 0.007 | 0.008 | 0.071 | 0.012 | 0.193 | 0.037 | 0.177 | 0.039 | 0.742 | 0.024 |
| 10 | 7 | 100 | 40 | 0.304 | 0.188 | 0.021 | 0.014 | 0.049 | 0.011 | 0.215 | 0.032 | 0.209 | 0.033 | 0.762 | 0.023 |

| | | | | | | | | | | | | | | | |
|---|---|---|---|---|---|---|---|---|---|---|---|---|---|---|---|
| 10 | 8 | 100 | 40 | 0.435 | 0.159 | 0.021 | 0.014 | 0.027 | 0.013 | 0.224 | 0.037 | 0.216 | 0.037 | 0.749 | 0.024 |
| 10 | 9 | 100 | 40 | 0.400 | 0.119 | 0.029 | 0.008 | 0.044 | 0.011 | 0.174 | 0.037 | 0.150 | 0.039 | 0.716 | 0.024 |
| 10 | 10 | 100 | 40 | 0.119 | 0.159 | 0.008 | 0.014 | 0.062 | 0.013 | 0.223 | 0.037 | 0.212 | 0.037 | 0.740 | 0.023 |
| 1 | 1 | 100 | 60 | 0.275 | 0.666 | 0.043 | 0.105 | 0.114 | 0.107 | 0.290 | 0.039 | 0.289 | 0.045 | 0.707 | 0.028 |
| 1 | 2 | 100 | 60 | 0.417 | 0.638 | 0.070 | 0.108 | 0.098 | 0.108 | 0.311 | 0.041 | 0.311 | 0.048 | 0.690 | 0.029 |
| 1 | 3 | 100 | 60 | 0.928 | 0.585 | 0.166 | 0.111 | 0.013 | 0.104 | 0.328 | 0.043 | 0.339 | 0.052 | 0.695 | 0.031 |
| 1 | 4 | 100 | 60 | 0.282 | 0.666 | 0.044 | 0.105 | 0.112 | 0.108 | 0.295 | 0.039 | 0.292 | 0.045 | 0.698 | 0.028 |
| 1 | 5 | 100 | 60 | 0.425 | 0.638 | 0.071 | 0.111 | 0.096 | 0.104 | 0.316 | 0.041 | 0.314 | 0.052 | 0.681 | 0.029 |
| 1 | 6 | 100 | 60 | 0.938 | 0.638 | 0.167 | 0.111 | 0.011 | 0.107 | 0.333 | 0.039 | 0.342 | 0.045 | 0.686 | 0.028 |
| 1 | 7 | 100 | 60 | 0.279 | 0.585 | 0.045 | 0.111 | 0.115 | 0.108 | 0.286 | 0.041 | 0.286 | 0.048 | 0.713 | 0.029 |
| 1 | 8 | 100 | 60 | 0.419 | 0.585 | 0.072 | 0.111 | 0.099 | 0.104 | 0.307 | 0.043 | 0.308 | 0.052 | 0.696 | 0.031 |
| 1 | 9 | 100 | 60 | 0.923 | 0.666 | 0.168 | 0.108 | 0.014 | 0.108 | 0.324 | 0.039 | 0.336 | 0.045 | 0.701 | 0.028 |
| 1 | 10 | 100 | 60 | 0.287 | 0.638 | 0.046 | 0.108 | 0.113 | 0.104 | 0.291 | 0.041 | 0.289 | 0.052 | 0.704 | 0.029 |
| 5 | 1 | 100 | 60 | 0.235 | 0.188 | 0.018 | 0.016 | 0.059 | 0.014 | 0.223 | 0.035 | 0.214 | 0.038 | 0.743 | 0.023 |
| 5 | 2 | 100 | 60 | 0.392 | 0.169 | 0.036 | 0.019 | 0.055 | 0.014 | 0.255 | 0.038 | 0.257 | 0.044 | 0.752 | 0.025 |
| 5 | 3 | 100 | 60 | 0.538 | 0.187 | 0.049 | 0.023 | 0.042 | 0.014 | 0.217 | 0.034 | 0.200 | 0.035 | 0.724 | 0.022 |
| 5 | 4 | 100 | 60 | 0.238 | 0.169 | 0.019 | 0.016 | 0.061 | 0.014 | 0.229 | 0.038 | 0.218 | 0.038 | 0.734 | 0.023 |
| 5 | 5 | 100 | 60 | 0.394 | 0.187 | 0.037 | 0.019 | 0.057 | 0.014 | 0.260 | 0.034 | 0.261 | 0.035 | 0.743 | 0.022 |
| 5 | 6 | 100 | 60 | 0.532 | 0.169 | 0.050 | 0.016 | 0.044 | 0.014 | 0.222 | 0.035 | 0.204 | 0.038 | 0.715 | 0.023 |
| 5 | 7 | 100 | 60 | 0.237 | 0.187 | 0.018 | 0.019 | 0.058 | 0.014 | 0.220 | 0.038 | 0.211 | 0.044 | 0.748 | 0.025 |
| 5 | 8 | 100 | 60 | 0.400 | 0.169 | 0.036 | 0.016 | 0.054 | 0.014 | 0.251 | 0.034 | 0.254 | 0.035 | 0.757 | 0.022 |
| 5 | 9 | 100 | 60 | 0.544 | 0.187 | 0.049 | 0.019 | 0.041 | 0.014 | 0.213 | 0.038 | 0.197 | 0.038 | 0.729 | 0.023 |
| 5 | 10 | 100 | 60 | 0.241 | 0.188 | 0.019 | 0.023 | 0.060 | 0.014 | 0.225 | 0.034 | 0.215 | 0.035 | 0.739 | 0.022 |
| 10 | 1 | 100 | 60 | 0.656 | 0.160 | 0.047 | 0.019 | 0.025 | 0.009 | 0.179 | 0.031 | 0.167 | 0.033 | 0.763 | 0.020 |
| 10 | 2 | 100 | 60 | 0.000 | 0.000 | 0.000 | 0.000 | 0.050 | 0.007 | 0.178 | 0.032 | 0.161 | 0.032 | 0.741 | 0.019 |
| 10 | 3 | 100 | 60 | 0.215 | 0.146 | 0.009 | 0.007 | 0.033 | 0.006 | 0.153 | 0.030 | 0.131 | 0.028 | 0.726 | 0.017 |
| 10 | 4 | 100 | 60 | 0.649 | 0.146 | 0.048 | 0.019 | 0.026 | 0.007 | 0.184 | 0.035 | 0.170 | 0.032 | 0.754 | 0.020 |
| 10 | 5 | 100 | 60 | 0.000 | 0.146 | 0.000 | 0.007 | 0.051 | 0.006 | 0.183 | 0.038 | 0.164 | 0.028 | 0.732 | 0.017 |
| 10 | 6 | 100 | 60 | 0.227 | 0.146 | 0.010 | 0.019 | 0.034 | 0.007 | 0.157 | 0.034 | 0.134 | 0.033 | 0.717 | 0.020 |
| 10 | 7 | 100 | 60 | 0.625 | 0.146 | 0.045 | 0.007 | 0.027 | 0.007 | 0.177 | 0.038 | 0.165 | 0.032 | 0.767 | 0.019 |
| 10 | 8 | 100 | 60 | 0.000 | 0.000 | 0.000 | 0.000 | 0.052 | 0.007 | 0.176 | 0.034 | 0.159 | 0.028 | 0.745 | 0.017 |
| 10 | 9 | 100 | 60 | 0.167 | 0.160 | 0.007 | 0.019 | 0.035 | 0.007 | 0.150 | 0.031 | 0.129 | 0.032 | 0.730 | 0.020 |
| 10 | 10 | 100 | 60 | 0.622 | 0.160 | 0.046 | 0.007 | 0.028 | 0.007 | 0.181 | 0.030 | 0.168 | 0.028 | 0.758 | 0.017 |
| 1 | 1 | 200 | 20 | 0.524 | 0.298 | 0.084 | 0.048 | 0.076 | 0.048 | 0.247 | 0.030 | 0.244 | 0.033 | 0.741 | 0.027 |
| 1 | 2 | 200 | 20 | 0.300 | 0.304 | 0.066 | 0.066 | 0.153 | 0.069 | 0.348 | 0.037 | 0.359 | 0.045 | 0.673 | 0.030 |
| 1 | 3 | 200 | 20 | 0.112 | 0.298 | 0.020 | 0.054 | 0.161 | 0.058 | 0.298 | 0.034 | 0.285 | 0.037 | 0.669 | 0.027 |
| 1 | 4 | 200 | 20 | 0.399 | 0.298 | 0.054 | 0.048 | 0.082 | 0.048 | 0.323 | 0.030 | 0.326 | 0.033 | 0.684 | 0.030 |
| 1 | 5 | 200 | 20 | 0.181 | 0.298 | 0.034 | 0.069 | 0.152 | 0.066 | 0.293 | 0.034 | 0.298 | 0.037 | 0.717 | 0.027 |
| 1 | 6 | 200 | 20 | 0.324 | 0.298 | 0.058 | 0.058 | 0.122 | 0.054 | 0.311 | 0.030 | 0.314 | 0.033 | 0.695 | 0.027 |
| 1 | 7 | 200 | 20 | 0.276 | 0.298 | 0.054 | 0.054 | 0.143 | 0.058 | 0.275 | 0.037 | 0.267 | 0.045 | 0.703 | 0.027 |
| 1 | 8 | 200 | 20 | 0.204 | 0.298 | 0.027 | 0.048 | 0.106 | 0.048 | 0.293 | 0.034 | 0.307 | 0.037 | 0.742 | 0.027 |
| 1 | 9 | 200 | 20 | 0.255 | 0.298 | 0.039 | 0.069 | 0.114 | 0.066 | 0.286 | 0.030 | 0.285 | 0.033 | 0.713 | 0.027 |
| 1 | 10 | 200 | 20 | 0.335 | 0.298 | 0.057 | 0.058 | 0.112 | 0.054 | 0.291 | 0.034 | 0.291 | 0.037 | 0.709 | 0.030 |
| 5 | 1 | 200 | 20 | 0.182 | 0.122 | 0.019 | 0.013 | 0.085 | 0.014 | 0.183 | 0.030 | 0.165 | 0.030 | 0.737 | 0.025 |
| 5 | 2 | 200 | 20 | 0.384 | 0.147 | 0.049 | 0.021 | 0.078 | 0.017 | 0.232 | 0.034 | 0.217 | 0.035 | 0.719 | 0.026 |
| 5 | 3 | 200 | 20 | 0.398 | 0.146 | 0.042 | 0.017 | 0.063 | 0.014 | 0.166 | 0.029 | 0.151 | 0.028 | 0.761 | 0.025 |
| 5 | 4 | 200 | 20 | 0.391 | 0.146 | 0.037 | 0.017 | 0.057 | 0.015 | 0.212 | 0.030 | 0.197 | 0.030 | 0.734 | 0.025 |

| | | | | | | | | | | | | | | | |
|---|---|---|---|---|---|---|---|---|---|---|---|---|---|---|---|
| 5 | 5 | 200 | 20 | 0.279 | 0.146 | 0.025 | 0.014 | 0.065 | 0.014 | 0.163 | 0.029 | 0.141 | 0.035 | 0.724 | 0.026 |
| 5 | 6 | 200 | 20 | 0.376 | 0.146 | 0.033 | 0.021 | 0.055 | 0.017 | 0.210 | 0.030 | 0.190 | 0.030 | 0.715 | 0.025 |
| 5 | 7 | 200 | 20 | 0.378 | 0.146 | 0.049 | 0.017 | 0.080 | 0.015 | 0.225 | 0.034 | 0.207 | 0.035 | 0.715 | 0.026 |
| 5 | 8 | 200 | 20 | 0.375 | 0.125 | 0.041 | 0.017 | 0.068 | 0.014 | 0.219 | 0.029 | 0.202 | 0.028 | 0.723 | 0.025 |
| 5 | 9 | 200 | 20 | 0.371 | 0.146 | 0.042 | 0.017 | 0.071 | 0.017 | 0.209 | 0.030 | 0.196 | 0.030 | 0.742 | 0.025 |
| 5 | 10 | 200 | 20 | 0.222 | 0.125 | 0.018 | 0.014 | 0.062 | 0.015 | 0.225 | 0.029 | 0.209 | 0.035 | 0.721 | 0.026 |
| 10 | 1 | 200 | 20 | 0.170 | 0.125 | 0.019 | 0.014 | 0.093 | 0.015 | 0.199 | 0.031 | 0.188 | 0.032 | 0.755 | 0.026 |
| 10 | 2 | 200 | 20 | 0.232 | 0.127 | 0.030 | 0.018 | 0.099 | 0.017 | 0.247 | 0.035 | 0.248 | 0.040 | 0.757 | 0.028 |
| 10 | 3 | 200 | 20 | 0.465 | 0.143 | 0.056 | 0.021 | 0.065 | 0.015 | 0.224 | 0.033 | 0.202 | 0.033 | 0.699 | 0.025 |
| 10 | 4 | 200 | 20 | 0.365 | 0.125 | 0.041 | 0.013 | 0.071 | 0.014 | 0.235 | 0.035 | 0.224 | 0.040 | 0.728 | 0.026 |
| 10 | 5 | 200 | 20 | 0.343 | 0.143 | 0.049 | 0.021 | 0.094 | 0.017 | 0.235 | 0.033 | 0.231 | 0.033 | 0.750 | 0.025 |
| 10 | 6 | 200 | 20 | 0.298 | 0.125 | 0.032 | 0.017 | 0.077 | 0.015 | 0.219 | 0.031 | 0.205 | 0.040 | 0.729 | 0.026 |
| 10 | 7 | 200 | 20 | 0.251 | 0.127 | 0.029 | 0.014 | 0.085 | 0.014 | 0.202 | 0.035 | 0.181 | 0.033 | 0.716 | 0.028 |
| 10 | 8 | 200 | 20 | 0.214 | 0.143 | 0.022 | 0.018 | 0.082 | 0.017 | 0.264 | 0.033 | 0.251 | 0.040 | 0.701 | 0.025 |
| 10 | 9 | 200 | 20 | 0.445 | 0.125 | 0.050 | 0.017 | 0.063 | 0.015 | 0.207 | 0.035 | 0.198 | 0.033 | 0.761 | 0.026 |
| 10 | 10 | 200 | 20 | 0.255 | 0.143 | 0.031 | 0.014 | 0.090 | 0.015 | 0.228 | 0.033 | 0.217 | 0.033 | 0.737 | 0.025 |
| 1 | 1 | 200 | 40 | 0.203 | 0.328 | 0.035 | 0.057 | 0.137 | 0.059 | 0.300 | 0.030 | 0.303 | 0.035 | 0.708 | 0.022 |
| 1 | 2 | 200 | 40 | 0.757 | 0.301 | 0.127 | 0.054 | 0.041 | 0.050 | 0.300 | 0.030 | 0.300 | 0.034 | 0.701 | 0.022 |
| 1 | 3 | 200 | 40 | 0.601 | 0.327 | 0.116 | 0.065 | 0.077 | 0.063 | 0.340 | 0.032 | 0.348 | 0.039 | 0.676 | 0.024 |
| 1 | 4 | 200 | 40 | 0.533 | 0.328 | 0.119 | 0.057 | 0.104 | 0.059 | 0.327 | 0.030 | 0.327 | 0.035 | 0.673 | 0.021 |
| 1 | 5 | 200 | 40 | 0.595 | 0.327 | 0.082 | 0.054 | 0.056 | 0.063 | 0.312 | 0.030 | 0.326 | 0.039 | 0.718 | 0.019 |
| 1 | 6 | 200 | 40 | 0.487 | 0.328 | 0.098 | 0.057 | 0.104 | 0.059 | 0.321 | 0.030 | 0.325 | 0.035 | 0.686 | 0.021 |
| 1 | 7 | 200 | 40 | 0.466 | 0.301 | 0.077 | 0.054 | 0.089 | 0.050 | 0.325 | 0.030 | 0.334 | 0.034 | 0.694 | 0.020 |
| 1 | 8 | 200 | 40 | 0.571 | 0.327 | 0.108 | 0.065 | 0.081 | 0.063 | 0.317 | 0.030 | 0.327 | 0.039 | 0.705 | 0.021 |
| 1 | 9 | 200 | 40 | 0.417 | 0.328 | 0.082 | 0.057 | 0.115 | 0.059 | 0.312 | 0.030 | 0.320 | 0.035 | 0.707 | 0.022 |
| 1 | 10 | 200 | 40 | 0.467 | 0.327 | 0.067 | 0.054 | 0.077 | 0.063 | 0.310 | 0.030 | 0.317 | 0.039 | 0.704 | 0.022 |
| 5 | 1 | 200 | 40 | 0.289 | 0.136 | 0.030 | 0.015 | 0.073 | 0.013 | 0.250 | 0.029 | 0.242 | 0.032 | 0.727 | 0.021 |
| 5 | 2 | 200 | 40 | 0.439 | 0.131 | 0.047 | 0.017 | 0.060 | 0.012 | 0.236 | 0.028 | 0.229 | 0.030 | 0.742 | 0.020 |
| 5 | 3 | 200 | 40 | 0.392 | 0.137 | 0.039 | 0.016 | 0.061 | 0.012 | 0.228 | 0.027 | 0.226 | 0.030 | 0.766 | 0.021 |
| 5 | 4 | 200 | 40 | 0.413 | 0.136 | 0.049 | 0.015 | 0.069 | 0.012 | 0.254 | 0.029 | 0.251 | 0.030 | 0.737 | 0.021 |
| 5 | 5 | 200 | 40 | 0.359 | 0.137 | 0.041 | 0.016 | 0.074 | 0.012 | 0.236 | 0.027 | 0.228 | 0.030 | 0.736 | 0.022 |
| 5 | 6 | 200 | 40 | 0.393 | 0.136 | 0.044 | 0.015 | 0.068 | 0.012 | 0.253 | 0.029 | 0.246 | 0.030 | 0.727 | 0.022 |
| 5 | 7 | 200 | 40 | 0.362 | 0.131 | 0.030 | 0.017 | 0.054 | 0.012 | 0.245 | 0.028 | 0.240 | 0.030 | 0.738 | 0.021 |
| 5 | 8 | 200 | 40 | 0.407 | 0.137 | 0.041 | 0.016 | 0.059 | 0.012 | 0.251 | 0.027 | 0.244 | 0.030 | 0.728 | 0.020 |
| 5 | 9 | 200 | 40 | 0.417 | 0.136 | 0.040 | 0.015 | 0.056 | 0.012 | 0.224 | 0.029 | 0.218 | 0.030 | 0.756 | 0.020 |
| 5 | 10 | 200 | 40 | 0.408 | 0.137 | 0.047 | 0.016 | 0.068 | 0.012 | 0.241 | 0.027 | 0.243 | 0.032 | 0.765 | 0.021 |
| 10 | 1 | 200 | 40 | 0.342 | 0.124 | 0.028 | 0.012 | 0.054 | 0.009 | 0.202 | 0.026 | 0.189 | 0.027 | 0.745 | 0.018 |
| 10 | 2 | 200 | 40 | 0.099 | 0.089 | 0.008 | 0.007 | 0.070 | 0.009 | 0.206 | 0.026 | 0.196 | 0.028 | 0.755 | 0.019 |
| 10 | 3 | 200 | 40 | 0.016 | 0.064 | 0.001 | 0.004 | 0.062 | 0.007 | 0.160 | 0.023 | 0.144 | 0.022 | 0.758 | 0.017 |
| 10 | 4 | 200 | 40 | 0.237 | 0.089 | 0.018 | 0.007 | 0.057 | 0.009 | 0.188 | 0.023 | 0.173 | 0.027 | 0.750 | 0.019 |
| 10 | 5 | 200 | 40 | 0.231 | 0.064 | 0.017 | 0.004 | 0.056 | 0.009 | 0.184 | 0.026 | 0.166 | 0.028 | 0.739 | 0.017 |
| 10 | 6 | 200 | 40 | 0.261 | 0.124 | 0.022 | 0.007 | 0.062 | 0.009 | 0.175 | 0.026 | 0.160 | 0.027 | 0.757 | 0.018 |
| 10 | 7 | 200 | 40 | 0.117 | 0.089 | 0.009 | 0.004 | 0.067 | 0.009 | 0.172 | 0.026 | 0.157 | 0.028 | 0.759 | 0.019 |
| 10 | 8 | 200 | 40 | 0.123 | 0.064 | 0.009 | 0.007 | 0.064 | 0.009 | 0.174 | 0.026 | 0.158 | 0.027 | 0.749 | 0.017 |
| 10 | 9 | 200 | 40 | 0.165 | 0.089 | 0.013 | 0.004 | 0.064 | 0.009 | 0.194 | 0.026 | 0.182 | 0.028 | 0.756 | 0.019 |
| 10 | 10 | 200 | 40 | 0.326 | 0.064 | 0.029 | 0.012 | 0.059 | 0.009 | 0.169 | 0.026 | 0.151 | 0.022 | 0.745 | 0.017 |
| 1 | 1 | 200 | 60 | 0.489 | 0.314 | 0.090 | 0.059 | 0.094 | 0.057 | 0.335 | 0.031 | 0.341 | 0.037 | 0.679 | 0.022 |

| | | | | | | | | | | | | | | | |
|---|---|---|---|---|---|---|---|---|---|---|---|---|---|---|---|
| 1 | 2 | 200 | 60 | 0.319 | 0.314 | 0.062 | 0.061 | 0.132 | 0.062 | 0.353 | 0.032 | 0.362 | 0.039 | 0.664 | 0.023 |
| 1 | 3 | 200 | 60 | 0.859 | 0.325 | 0.146 | 0.060 | 0.024 | 0.055 | 0.316 | 0.029 | 0.313 | 0.034 | 0.679 | 0.020 |
| 1 | 4 | 200 | 60 | 0.529 | 0.314 | 0.088 | 0.060 | 0.079 | 0.057 | 0.312 | 0.031 | 0.349 | 0.037 | 0.770 | 0.022 |
| 1 | 5 | 200 | 60 | 0.724 | 0.314 | 0.094 | 0.060 | 0.036 | 0.055 | 0.303 | 0.029 | 0.335 | 0.034 | 0.772 | 0.020 |
| 1 | 6 | 200 | 60 | 0.642 | 0.314 | 0.109 | 0.060 | 0.061 | 0.057 | 0.302 | 0.032 | 0.334 | 0.037 | 0.771 | 0.022 |
| 1 | 7 | 200 | 60 | 0.391 | 0.314 | 0.081 | 0.060 | 0.126 | 0.062 | 0.300 | 0.029 | 0.331 | 0.034 | 0.771 | 0.023 |
| 1 | 8 | 200 | 60 | 0.504 | 0.314 | 0.119 | 0.060 | 0.117 | 0.055 | 0.296 | 0.031 | 0.325 | 0.039 | 0.771 | 0.020 |
| 1 | 9 | 200 | 60 | 0.887 | 0.314 | 0.104 | 0.060 | 0.013 | 0.057 | 0.312 | 0.032 | 0.349 | 0.037 | 0.770 | 0.022 |
| 1 | 10 | 200 | 60 | 0.468 | 0.314 | 0.091 | 0.060 | 0.104 | 0.055 | 0.315 | 0.029 | 0.354 | 0.034 | 0.771 | 0.020 |
| 5 | 1 | 200 | 60 | 0.331 | 0.114 | 0.022 | 0.009 | 0.044 | 0.006 | 0.203 | 0.024 | 0.185 | 0.025 | 0.725 | 0.014 |
| 5 | 2 | 200 | 60 | 0.279 | 0.115 | 0.019 | 0.009 | 0.049 | 0.008 | 0.188 | 0.022 | 0.174 | 0.023 | 0.749 | 0.015 |
| 5 | 3 | 200 | 60 | 0.316 | 0.127 | 0.026 | 0.012 | 0.057 | 0.010 | 0.229 | 0.025 | 0.217 | 0.027 | 0.728 | 0.017 |
| 5 | 4 | 200 | 60 | 0.311 | 0.114 | 0.024 | 0.009 | 0.053 | 0.006 | 0.198 | 0.025 | 0.182 | 0.027 | 0.737 | 0.014 |
| 5 | 5 | 200 | 60 | 0.352 | 0.114 | 0.021 | 0.009 | 0.039 | 0.008 | 0.200 | 0.026 | 0.184 | 0.026 | 0.737 | 0.016 |
| 5 | 6 | 200 | 60 | 0.359 | 0.114 | 0.024 | 0.009 | 0.043 | 0.006 | 0.226 | 0.024 | 0.215 | 0.025 | 0.736 | 0.016 |
| 5 | 7 | 200 | 60 | 0.247 | 0.114 | 0.018 | 0.009 | 0.055 | 0.008 | 0.206 | 0.022 | 0.191 | 0.023 | 0.737 | 0.014 |
| 5 | 8 | 200 | 60 | 0.329 | 0.114 | 0.023 | 0.009 | 0.046 | 0.010 | 0.211 | 0.025 | 0.197 | 0.027 | 0.737 | 0.015 |
| 5 | 9 | 200 | 60 | 0.314 | 0.114 | 0.022 | 0.009 | 0.047 | 0.006 | 0.233 | 0.025 | 0.225 | 0.027 | 0.739 | 0.015 |
| 5 | 10 | 200 | 60 | 0.272 | 0.114 | 0.023 | 0.009 | 0.062 | 0.008 | 0.205 | 0.026 | 0.190 | 0.026 | 0.737 | 0.017 |
| 10 | 1 | 200 | 60 | 0.359 | 0.112 | 0.022 | 0.009 | 0.040 | 0.006 | 0.217 | 0.025 | 0.201 | 0.027 | 0.726 | 0.014 |
| 10 | 2 | 200 | 60 | 0.524 | 0.114 | 0.044 | 0.014 | 0.040 | 0.007 | 0.242 | 0.026 | 0.229 | 0.029 | 0.715 | 0.016 |
| 10 | 3 | 200 | 60 | 0.195 | 0.121 | 0.013 | 0.009 | 0.055 | 0.008 | 0.214 | 0.024 | 0.202 | 0.026 | 0.740 | 0.016 |
| 10 | 4 | 200 | 60 | 0.420 | 0.114 | 0.023 | 0.009 | 0.032 | 0.006 | 0.234 | 0.024 | 0.218 | 0.025 | 0.716 | 0.014 |
| 10 | 5 | 200 | 60 | 0.432 | 0.114 | 0.037 | 0.009 | 0.049 | 0.008 | 0.227 | 0.022 | 0.217 | 0.023 | 0.737 | 0.015 |
| 10 | 6 | 200 | 60 | 0.493 | 0.114 | 0.038 | 0.009 | 0.039 | 0.006 | 0.226 | 0.025 | 0.212 | 0.027 | 0.730 | 0.014 |
| 10 | 7 | 200 | 60 | 0.388 | 0.114 | 0.039 | 0.009 | 0.062 | 0.008 | 0.228 | 0.022 | 0.215 | 0.027 | 0.728 | 0.016 |
| 10 | 8 | 200 | 60 | 0.466 | 0.114 | 0.039 | 0.009 | 0.045 | 0.007 | 0.234 | 0.025 | 0.225 | 0.026 | 0.737 | 0.016 |
| 10 | 9 | 200 | 60 | 0.399 | 0.114 | 0.037 | 0.009 | 0.057 | 0.008 | 0.228 | 0.025 | 0.214 | 0.029 | 0.725 | 0.014 |
| 10 | 10 | 200 | 60 | 0.296 | 0.114 | 0.027 | 0.009 | 0.065 | 0.006 | 0.225 | 0.026 | 0.208 | 0.026 | 0.717 | 0.015 |
| 1 | 1 | 400 | 20 | 0.657 | 0.289 | 0.168 | 0.039 | 0.283 | 0.039 | 0.175 | 0.021 | 0.165 | 0.024 | 0.776 | 0.018 |
| 1 | 2 | 400 | 20 | 0.305 | 0.295 | 0.022 | 0.057 | 0.695 | 0.060 | 0.414 | 0.028 | 0.432 | 0.036 | 0.612 | 0.021 |
| 1 | 3 | 400 | 20 | 0.564 | 0.289 | 0.004 | 0.045 | 0.439 | 0.049 | 0.324 | 0.025 | 0.312 | 0.028 | 0.651 | 0.018 |
| 1 | 4 | 400 | 20 | 0.197 | 0.289 | 0.108 | 0.039 | 0.439 | 0.039 | 0.354 | 0.021 | 0.349 | 0.024 | 0.636 | 0.021 |
| 1 | 5 | 400 | 20 | 0.132 | 0.289 | 0.087 | 0.060 | 0.573 | 0.057 | 0.316 | 0.025 | 0.323 | 0.028 | 0.698 | 0.018 |
| 1 | 6 | 400 | 20 | 0.126 | 0.289 | 0.070 | 0.049 | 0.487 | 0.045 | 0.377 | 0.021 | 0.392 | 0.024 | 0.649 | 0.018 |
| 1 | 7 | 400 | 20 | 0.254 | 0.289 | 0.108 | 0.045 | 0.318 | 0.049 | 0.269 | 0.028 | 0.243 | 0.036 | 0.662 | 0.018 |
| 1 | 8 | 400 | 20 | 0.143 | 0.289 | 0.099 | 0.039 | 0.592 | 0.039 | 0.299 | 0.025 | 0.330 | 0.028 | 0.774 | 0.018 |
| 1 | 9 | 400 | 20 | 0.153 | 0.289 | 0.077 | 0.060 | 0.427 | 0.057 | 0.277 | 0.021 | 0.260 | 0.024 | 0.677 | 0.018 |
| 1 | 10 | 400 | 20 | 0.131 | 0.289 | 0.067 | 0.049 | 0.443 | 0.045 | 0.295 | 0.025 | 0.288 | 0.028 | 0.687 | 0.021 |
| 5 | 1 | 400 | 20 | 0.162 | 0.113 | 0.038 | 0.004 | 0.197 | 0.005 | 0.072 | 0.021 | 0.055 | 0.021 | 0.705 | 0.016 |
| 5 | 2 | 400 | 20 | 0.516 | 0.138 | 0.042 | 0.012 | 0.355 | 0.008 | 0.210 | 0.025 | 0.191 | 0.026 | 0.717 | 0.017 |
| 5 | 3 | 400 | 20 | 0.415 | 0.137 | 0.073 | 0.008 | 0.217 | 0.005 | 0.175 | 0.020 | 0.161 | 0.019 | 0.757 | 0.016 |
| 5 | 4 | 400 | 20 | 0.260 | 0.137 | 0.074 | 0.008 | 0.262 | 0.006 | 0.155 | 0.021 | 0.130 | 0.021 | 0.707 | 0.016 |
| 5 | 5 | 400 | 20 | 0.047 | 0.137 | 0.010 | 0.005 | 0.201 | 0.005 | 0.083 | 0.020 | 0.065 | 0.026 | 0.715 | 0.017 |
| 5 | 6 | 400 | 20 | 0.154 | 0.137 | 0.054 | 0.012 | 0.296 | 0.008 | 0.163 | 0.021 | 0.130 | 0.021 | 0.667 | 0.016 |
| 5 | 7 | 400 | 20 | 0.228 | 0.137 | 0.098 | 0.008 | 0.332 | 0.006 | 0.173 | 0.025 | 0.142 | 0.026 | 0.680 | 0.017 |
| 5 | 8 | 400 | 20 | 0.080 | 0.116 | 0.024 | 0.008 | 0.275 | 0.005 | 0.192 | 0.020 | 0.166 | 0.019 | 0.699 | 0.016 |

| | | | | | | | | | | | | | | | |
|---|---|---|---|---|---|---|---|---|---|---|---|---|---|---|---|
| 5 | 9 | 400 | 20 | 0.170 | 0.137 | 0.063 | 0.008 | 0.308 | 0.008 | 0.177 | 0.021 | 0.157 | 0.021 | 0.731 | 0.016 |
| 5 | 10 | 400 | 20 | 0.056 | 0.116 | 0.017 | 0.005 | 0.286 | 0.006 | 0.210 | 0.020 | 0.180 | 0.026 | 0.676 | 0.017 |
| 10 | 1 | 400 | 20 | 0.377 | 0.116 | 0.024 | 0.005 | 0.277 | 0.006 | 0.173 | 0.022 | 0.159 | 0.023 | 0.762 | 0.017 |
| 10 | 2 | 400 | 20 | 0.435 | 0.118 | 0.038 | 0.009 | 0.399 | 0.008 | 0.242 | 0.026 | 0.256 | 0.031 | 0.804 | 0.019 |
| 10 | 3 | 400 | 20 | 0.345 | 0.134 | 0.112 | 0.012 | 0.253 | 0.006 | 0.061 | 0.024 | 0.048 | 0.024 | 0.743 | 0.016 |
| 10 | 4 | 400 | 20 | 0.154 | 0.116 | 0.065 | 0.004 | 0.356 | 0.005 | 0.173 | 0.026 | 0.159 | 0.031 | 0.761 | 0.017 |
| 10 | 5 | 400 | 20 | 0.169 | 0.134 | 0.074 | 0.012 | 0.364 | 0.008 | 0.185 | 0.024 | 0.177 | 0.024 | 0.782 | 0.016 |
| 10 | 6 | 400 | 20 | 0.172 | 0.116 | 0.049 | 0.008 | 0.236 | 0.006 | 0.105 | 0.022 | 0.083 | 0.031 | 0.704 | 0.017 |
| 10 | 7 | 400 | 20 | 0.232 | 0.118 | 0.058 | 0.005 | 0.192 | 0.005 | 0.135 | 0.026 | 0.108 | 0.024 | 0.694 | 0.019 |
| 10 | 8 | 400 | 20 | 0.045 | 0.134 | 0.019 | 0.009 | 0.404 | 0.008 | 0.243 | 0.024 | 0.213 | 0.031 | 0.665 | 0.016 |
| 10 | 9 | 400 | 20 | 0.249 | 0.116 | 0.100 | 0.008 | 0.301 | 0.006 | 0.084 | 0.026 | 0.073 | 0.024 | 0.801 | 0.017 |
| 10 | 10 | 400 | 20 | 0.131 | 0.134 | 0.051 | 0.005 | 0.339 | 0.006 | 0.194 | 0.024 | 0.188 | 0.024 | 0.779 | 0.016 |
