# Supplementary material for: Estimating heritability using family-pooled phenotypic and genotypic data: a simulation study applied to aquaculture
Source: Heredity (Edinb). 2022 Jan 31;128(3):178–86. doi: 10.1038/s41437-022-00502-8 (PMC8897491; doi:10.1038/s41437-022-00502-8)
Supplement: Supplementary file 2 — S2_Heritability of family means (pool) and pedigree (ped) Heritability, their associated additive (σa²), residual (σe²) variance and respective standard erros (SE). [file 41437_2022_502_MOESM2_ESM.pdf]

Supplementary Table S2. Heritability of family means (pool) and pedigree (ped) Heritability, their associated additive (σ_a^2), residual (σ_e^2) variance and respective standard erros (SE). The results are for two- and five-pool Scenario and for trait heritability of 0.3. For 200 family/ 60 progeny using 2 pools, repeatability (t), interclass corelation (ICC) and family mean heritability using Falconers' formula (falc) are provided across trait heritabilities of 0.05, 0.3 and 0.5.

Generation	Replicate	No. Families	Family Size	Trait Heritability	No. Pools	h^2(pool)	SE	σ_a^2 (pool)	SE	σ_e^2 (pool)	SE	h^2(ped)	SE	σ_a^2 (ped)	SE	σ_e^2 (ped)	SE	t	SE	ICC	h^2(falc)
1	1	100	20	0.3	2	0.754	0.058	0.171	0.030	0.056	0.011	0.313	0.049	0.323	0.058	0.706	0.041	NA	NA	NA	NA
1	2	100	20	0.3	2	0.797	0.053	0.160	0.027	0.041	0.009	0.280	0.046	0.286	0.053	0.734	0.040	NA	NA	NA	NA
1	3	100	20	0.3	2	0.676	0.068	0.131	0.025	0.063	0.011	0.273	0.045	0.258	0.048	0.687	0.037	NA	NA	NA	NA
1	4	100	20	0.3	2	0.718	0.058	0.168	0.027	0.066	0.011	0.293	0.046	0.303	0.056	0.732	0.042	NA	NA	NA	NA
1	5	100	20	0.3	2	0.700	0.053	0.148	0.025	0.064	0.009	0.271	0.045	0.273	0.052	0.736	0.035	NA	NA	NA	NA
1	6	100	20	0.3	2	0.723	0.058	0.147	0.030	0.056	0.011	0.241	0.043	0.236	0.046	0.741	0.038	NA	NA	NA	NA
1	7	100	20	0.3	2	0.766	0.053	0.129	0.027	0.039	0.011	0.281	0.046	0.291	0.051	0.744	0.039	NA	NA	NA	NA
1	8	100	20	0.3	2	0.702	0.068	0.135	0.025	0.057	0.011	0.286	0.045	0.284	0.053	0.710	0.041	NA	NA	NA	NA
1	9	100	20	0.3	2	0.765	0.058	0.181	0.027	0.056	0.009	0.293	0.048	0.310	0.056	0.749	0.036	NA	NA	NA	NA
1	10	100	20	0.3	2	0.709	0.053	0.159	0.025	0.065	0.011	0.287	0.045	0.294	0.050	0.731	0.040	NA	NA	NA	NA
5	1	100	20	0.3	2	0.465	0.109	0.094	0.030	0.108	0.018	0.263	0.052	0.275	0.062	0.769	0.043	NA	NA	NA	NA
5	2	100	20	0.3	2	0.468	0.097	0.088	0.025	0.100	0.016	0.252	0.049	0.243	0.054	0.721	0.039	NA	NA	NA	NA
5	3	100	20	0.3	2	0.279	0.107	0.043	0.019	0.110	0.016	0.156	0.040	0.141	0.038	0.765	0.034	NA	NA	NA	NA
5	4	100	20	0.3	2	0.437	0.109	0.082	0.025	0.106	0.016	0.258	0.050	0.264	0.062	0.761	0.042	NA	NA	NA	NA
5	5	100	20	0.3	2	0.409	0.107	0.073	0.019	0.105	0.016	0.228	0.047	0.217	0.055	0.733	0.039	NA	NA	NA	NA
5	6	100	20	0.3	2	0.368	0.109	0.059	0.030	0.102	0.018	0.246	0.042	0.250	0.037	0.763	0.035	NA	NA	NA	NA
5	7	100	20	0.3	2	0.419	0.097	0.078	0.025	0.108	0.016	0.266	0.051	0.272	0.028	0.750	0.031	NA	NA	NA	NA
5	8	100	20	0.3	2	0.396	0.107	0.082	0.019	0.125	0.016	0.242	0.049	0.238	0.064	0.747	0.039	NA	NA	NA	NA
5	9	100	20	0.3	2	0.330	0.109	0.054	0.025	0.110	0.016	0.238	0.045	0.235	0.052	0.753	0.038	NA	NA	NA	NA
5	10	100	20	0.3	2	0.488	0.107	0.105	0.019	0.110	0.016	0.237	0.052	0.238	0.035	0.766	0.033	NA	NA	NA	NA
10	1	100	20	0.3	2	0.473	0.106	0.081	0.026	0.090	0.014	0.225	0.049	0.217	0.053	0.748	0.038	NA	NA	NA	NA
10	2	100	20	0.3	2	0.591	0.089	0.105	0.027	0.072	0.012	0.253	0.051	0.240	0.055	0.710	0.037	NA	NA	NA	NA
10	3	100	20	0.3	2	0.697	0.071	0.169	0.037	0.074	0.013	0.352	0.058	0.356	0.071	0.655	0.042	NA	NA	NA	NA
10	4	100	20	0.3	2	0.565	0.106	0.110	0.026	0.084	0.014	0.294	0.043	0.289	0.056	0.695	0.033	NA	NA	NA	NA
10	5	100	20	0.3	2	0.615	0.089	0.126	0.037	0.079	0.013	0.284	0.045	0.285	0.058	0.718	0.035	NA	NA	NA	NA
10	6	100	20	0.3	2	0.542	0.089	0.104	0.026	0.088	0.014	0.303	0.047	0.327	0.060	0.754	0.037	NA	NA	NA	NA
10	7	100	20	0.3	2	0.600	0.071	0.113	0.027	0.075	0.012	0.256	0.049	0.254	0.062	0.738	0.039	NA	NA	NA	NA
10	8	100	20	0.3	2	0.605	0.106	0.137	0.037	0.089	0.013	0.282	0.051	0.289	0.064	0.737	0.041	NA	NA	NA	NA
10	9	100	20	0.3	2	0.620	0.071	0.122	0.026	0.075	0.014	0.310	0.053	0.323	0.066	0.721	0.043	NA	NA	NA	NA
10	10	100	20	0.3	2	0.564	0.106	0.103	0.037	0.080	0.013	0.261	0.055	0.246	0.068	0.695	0.045	NA	NA	NA	NA
1	1	100	40	0.3	2	0.768	0.050	0.134	0.023	0.041	0.007	0.263	0.038	0.261	0.043	0.731	0.029	NA	NA	NA	NA
1	2	100	40	0.3	2	0.822	0.041	0.142	0.023	0.031	0.006	0.281	0.040	0.263	0.043	0.673	0.029	NA	NA	NA	NA
1	3	100	40	0.3	2	0.827	0.040	0.171	0.028	0.036	0.007	0.316	0.043	0.321	0.052	0.693	0.033	NA	NA	NA	NA
1	4	100	40	0.3	2	0.766	0.041	0.134	0.023	0.041	0.007	0.277	0.038	0.279	0.043	0.730	0.029	NA	NA	NA	NA
1	5	100	40	0.3	2	0.821	0.040	0.142	0.028	0.031	0.006	0.296	0.040	0.291	0.052	0.693	0.033	NA	NA	NA	NA
1	6	100	40	0.3	2	0.828	0.050	0.173	0.023	0.036	0.007	0.300	0.038	0.313	0.043	0.729	0.029	NA	NA	NA	NA
1	7	100	40	0.3	2	0.788	0.041	0.134	0.023	0.036	0.007	0.290	0.040	0.291	0.043	0.713	0.029	NA	NA	NA	NA
1	8	100	40	0.3	2	0.775	0.040	0.141	0.028	0.041	0.006	0.283	0.043	0.278	0.052	0.703	0.033	NA	NA	NA	NA
1	9	100	40	0.3	2	0.847	0.041	0.172	0.023	0.031	0.007	0.296	0.038	0.309	0.043	0.734	0.029	NA	NA	NA	NA
1	10	100	40	0.3	2	0.791	0.040	0.136	0.028	0.036	0.007	0.307	0.040	0.301	0.052	0.679	0.033	NA	NA	NA	NA
5	1	100	40	0.3	2	0.702	0.065	0.084	0.018	0.036	0.006	0.202	0.036	0.188	0.037	0.741	0.026	NA	NA	NA	NA
5	2	100	40	0.3	2	0.772	0.055	0.145	0.030	0.043	0.007	0.300	0.045	0.308	0.055	0.720	0.032	NA	NA	NA	NA
5	3	100	40	0.3	2	0.507	0.098	0.051	0.015	0.050	0.007	0.162	0.032	0.143	0.030	0.737	0.023	NA	NA	NA	NA
5	4	100	40	0.3	2	0.771	0.065	0.145	0.018	0.043	0.007	0.196	0.036	0.178	0.037	0.730	0.026	NA	NA	NA	NA
5	5	100	40	0.3	2	0.505	0.055	0.051	0.030	0.050	0.007	0.234	0.032	0.228	0.030	0.746	0.023	NA	NA	NA	NA
5	6	100	40	0.3	2	0.700	0.098	0.084	0.015	0.036	0.006	0.199	0.036	0.182	0.037	0.732	0.026	NA	NA	NA	NA
5	7	100	40	0.3	2	0.771	0.098	0.145	0.018	0.043	0.007	0.195	0.045	0.177	0.055	0.729	0.032	NA	NA	NA	NA
5	8	100	40	0.3	2	0.505	0.065	0.051	0.030	0.050	0.007	0.223	0.032	0.210	0.030	0.732	0.023	NA	NA	NA	NA
5	9	100	40	0.3	2	0.771	0.055	0.145	0.015	0.043	0.006	0.245	0.036	0.232	0.037	0.715	0.026	NA	NA	NA	NA
5	10	100	40	0.3	2	0.505	0.098	0.051	0.015	0.050	0.006	0.227	0.032	0.217	0.030	0.738	0.023	NA	NA	NA	NA
10	1	100	40	0.3	2	0.575	0.091	0.064	0.018	0.047	0.007	0.201	0.037	0.185	0.039	0.735	0.024	NA	NA	NA	NA
10	2	100	40	0.3	2	0.582	0.086	0.059	0.016	0.042	0.006	0.158	0.032	0.147	0.033	0.781	0.023	NA	NA	NA	NA
10	3	100	40	0.3	2	0.557	0.092	0.064	0.018	0.051	0.008	0.205	0.037	0.184	0.037	0.714	0.024	NA	NA	NA	NA
10	4	100	40	0.3	2	0.578	0.086	0.059	0.016	0.043	0.007	0.138	0.037	0.128	0.039	0.798	0.024	NA	NA	NA	NA
10	5	100	40	0.3	2	0.561	0.092	0.064	0.018	0.050	0.007	0.185	0.037	0.165	0.037	0.730	0.023	NA	NA	NA	NA
10	6	100	40	0.3	2	0.598	0.091	0.064	0.016	0.043	0.006	0.193	0.037	0.177	0.039	0.742	0.024	NA	NA	NA	NA
10	7	100	40	0.3	2	0.541	0.086	0.059	0.018	0.050	0.006	0.215	0.032	0.209	0.033	0.762	0.023	NA	NA	NA	NA
10	8	100	40	0.3	2	0.577	0.092	0.064	0.016	0.047	0.007	0.224	0.037	0.216	0.037	0.749	0.024	NA	NA	NA	NA
10	9	100	40	0.3	2	0.584	0.086	0.059	0.018	0.042	0.006	0.174	0.037	0.150	0.039	0.716	0.024	NA	NA	NA	NA
10	10	100	40	0.3	2	0.557	0.092	0.064	0.018	0.051	0.008	0.223	0.037	0.212	0.037	0.740	0.023	NA	NA	NA	NA
1	1	100	60	0.3	2	0.868	0.030	0.149	0.023	0.023	0.004	0.290	0.039	0.289	0.045	0.707	0.028	NA	NA	NA	NA
1	2	100	60	0.3	2	0.876	0.029	0.159	0.025	0.023	0.004	0.311	0.041	0.311	0.048	0.690	0.029	NA	NA	NA	NA
1	3	100	60	0.3	2	0.857	0.032	0.165	0.026	0.028	0.005	0.328	0.043	0.339	0.052	0.695	0.031	NA	NA	NA	NA
1	4	100	60	0.3	2	0.874	0.029	0.159	0.023	0.023	0.004	0.295	0.039	0.292	0.045	0.698	0.028	NA	NA	NA	NA
1	5	100	60	0.3	2	0.874	0.029	0.159	0.025	0.023	0.004	0.316	0.041	0.314	0.052	0.681	0.029	NA	NA	NA	NA
1	6	100	60	0.3	2	0.850	0.029	0.159	0.025	0.028	0.004	0.333	0.039	0.342	0.045	0.686	0.028	NA	NA	NA	NA
1	7	100	60	0.3	2	0.866	0.029	0.149	0.025	0.023	0.004	0.286	0.041	0.286	0.048	0.713	0.029	NA	NA	NA	NA
1	8	100	60	0.3	2	0.866	0.030	0.149	0.025	0.023	0.005	0.307	0.043	0.308	0.052	0.696	0.031	NA	NA	NA	NA
1	9	100	60	0.3	2	0.866	0.032	0.149	0.023	0.023	0.004	0.324	0.039	0.336	0.045	0.701	0.028	NA	NA	NA	NA
1	10	100	60	0.3	2	0.866	0.032	0.149	0.025	0.023	0.004	0.291	0.041	0.289	0.052	0.704	0.029	NA	NA	NA	NA
5	1	100	60	0.3	2	0.858	0.034	0.113	0.020	0.019	0.003	0.223	0.035	0.214	0.038	0.743	0.023	NA	NA	NA	NA
5	2	100	60	0.3	2	0.828	0.043	0.128	0.025	0.027	0.004	0.255	0.038	0.257	0.044	0.752	0.025	NA	NA	NA	NA
5	3	100	60	0.3	2	0.734	0.059	0.093	0.020	0.034	0.005	0.217	0.034	0.200	0.035	0.724	0.022	NA	NA	NA	NA
5	4	100	60	0.3	2	0.775	0.043	0.093	0.020	0.027	0.004	0.229	0.038	0.218	0.038	0.734	0.023	NA	NA	NA	NA
5	5	100	60	0.3	2	0.775	0.043	0.093	0.020	0.027	0.004	0.260	0.034	0.261	0.035	0.743	0.022	NA	NA	NA	NA
5	6	100	60	0.3	2	0.826	0.043	0.128	0.020	0.027	0.004	0.222	0.035	0.204	0.038	0.715	0.023	NA	NA	NA	NA
5	7	100	60	0.3	2	0.807	0.034	0.113	0.020	0.027	0.004	0.220	0.038	0.211	0.044	0.748	0.025	NA	NA	NA	NA
5	8	100	60	0.3	2	0.769	0.034	0.113	0.020	0.034	0.004	0.251	0.034	0.254	0.035	0.757	0.022	NA	NA	NA	NA
5	9	100	60	0.3	2	0.856	0.034	0.113	0.020	0.019	0.004	0.213	0.038	0.197	0.038	0.729	0.023	NA	NA	NA	NA
5	10	100	60	0.3	2	0.856	0.043	0.113	0.020	0.019	0.004	0.225	0.034	0.215	0.035	0.739	0.022	NA	NA	NA	NA
10	1	100	60	0.3	2	0.716	0.064	0.072	0.016	0.028	0.004	0.179	0.031	0.167	0.033	0.763	0.020	NA	NA	NA	NA
10	2	100	60	0.3	2	0.539	0.098	0.045	0.014	0.039	0.005	0.178	0.032	0.161	0.032	0.741	0.019	NA	NA	NA	NA
10	3	100	60	0.3	2	0.410	0.108	0.026	0.010	0.038	0.005	0.153	0.030	0.131	0.028	0.726	0.017	NA	NA	NA	NA
10	4	100	60	0.3	2	0.720	0.098	0.072	0.014	0.028	0.005	0.184	0.035	0.170	0.032	0.754	0.020	NA	NA	NA	NA
10	5	100	60	0.3	2	0.536	0.098	0.045	0.010	0.039	0.005	0.183	0.038	0.164	0.028	0.732	0.017	NA	NA	NA	NA
10	6	100	60	0.3	2	0.406	0.108	0.026	0.016	0.038	0.005	0.157	0.034	0.134	0.033	0.717	0.020	NA	NA	NA	NA
10	7	100	60	0.3	2	0.655	0.064	0.072	0.016	0.038	0.005	0.177	0.038	0.165	0.032	0.767	0.019	NA	NA	NA	NA
10	8	100	60	0.3	2	0.542	0.064	0.045	0.016	0.038	0.005	0.176	0.034	0.159	0.028	0.745	0.017	NA	NA	NA	NA
10	9	100	60	0.3	2	0.481	0.064	0.026	0.014	0.028	0.005	0.150	0.031	0.129	0.032	0.730	0.020	NA	NA	NA	NA
10	10	100	60	0.3	2	0.400	0.064	0.026	0.010	0.039	0.005	0.181	0.030	0.168	0.028	0.758	0.017	NA	NA	NA	NA
1	1	200	20	0.3	2	0.630	0.051	0.122	0.017	0.072	0.009	0.247	0.030	0.244	0.033	0.741	0.027	NA	NA	NA	NA
1	2	200	20	0.3	2	0.727	0.043	0.183	0.023	0.069	0.009	0.348	0.037	0.359	0.045	0.673	0.030	NA	NA	NA	NA
1	3	200	20	0.3	2	0.670	0.049	0.145	0.020	0.072	0.009	0.298	0.034	0.285	0.037	0.669	0.027	NA	NA	NA	NA
1	4	200	20	0.3	2	0.720	0.043	0.179	0.017	0.069	0.009	0.323	0.030	0.326	0.033	0.684	0.030	NA	NA	NA	NA
1	5	200	20	0.3	2	0.675	0.049	0.150	0.016	0.072	0.009	0.293	0.034	0.298	0.037	0.717	0.027	NA	NA	NA	NA
1	6	200	20	0.3	2	0.757	0.051	0.167	0.019	0.054	0.009	0.311	0.030	0.314	0.033	0.695	0.027	NA	NA	NA	NA
1	7	200	20	0.3	2	0.675	0.043	0.150	0.015	0.072	0.009	0.275	0.037	0.267	0.045	0.703	0.027	NA	NA	NA	NA
1	8	200	20	0.3	2	0.694	0.049	0.160	0.018	0.071	0.009	0.293	0.034	0.307	0.037	0.742	0.027	NA	NA	NA	NA
1	9	200	20	0.3	2	0.700	0.043	0.163	0.020	0.070	0.009	0.286	0.030	0.285	0.033	0.713	0.027	NA	NA	NA	NA
1	10	200	20	0.3	2	0.673	0.049	0.148	0.017	0.072	0.009	0.291	0.034	0.291	0.037	0.709	0.030	NA	NA	NA	NA
5	1	200	20	0.3	2	0.472	0.072	0.073	0.016	0.082	0.009	0.183	0.030	0.165	0.030	0.737	0.025	NA	NA	NA	NA
5	2	200	20	0.3	2	0.544	0.065	0.098	0.019	0.082	0.009	0.232	0.034	0.217	0.035	0.719	0.026	NA	NA	NA	NA
5	3	200	20	0.3	2	0.519	0.066	0.077	0.015	0.071	0.008	0.166	0.029	0.151	0.028	0.761	0.025	NA	NA	NA	NA
5	4	200	20	0.3	2	0.521	0.073	0.083	0.018	0.076	0.009	0.212	0.030	0.197	0.030	0.734	0.025	NA	NA	NA	NA
5	5	200	20	0.3	2	0.541	0.072	0.089	0.017	0.075	0.009	0.163	0.029	0.141	0.035	0.724	0.026	NA	NA	NA	NA
5	6	200	20	0.3	2	0.583	0.063	0.089	0.016	0.064	0.009	0.210	0.030	0.190	0.030	0.715	0.025	NA	NA	NA	NA
5	7	200	20	0.3	2	0.439	0.072	0.066	0.019	0.085	0.009	0.225	0.034	0.207	0.035	0.715	0.026	NA	NA	NA	NA
5	8	200	20	0.3	2	0.521	0.065	0.079	0.015	0.072	0.009	0.219	0.029	0.202	0.028	0.723	0.025	NA	NA	NA	NA
5	9	200	20	0.3	2	0.510	0.066	0.083	0.018	0.080	0.009	0.209	0.030	0.196	0.030	0.742	0.025	NA	NA	NA	NA
5	10	200	20	0.3	2	0.538	0.066	0.085	0.016	0.073	0.009	0.225	0.029	0.209	0.035	0.721	0.026	NA	NA	NA	NA
10	1	200	20	0.3	2	0.415	0.073	0.069	0.016	0.098	0.010	0.199	0.031	0.188	0.032	0.755	0.026	NA	NA	NA	NA

10	2	200	20	0.3	2	0.416	0.072	0.080	0.019	0.112	0.012	0.247	0.035	0.248	0.040	0.757	0.028	NA	NA	NA	NA
10	3	200	20	0.3	2	0.555	0.063	0.095	0.018	0.076	0.008	0.224	0.033	0.202	0.033	0.699	0.025	NA	NA	NA	NA
10	4	200	20	0.3	2	0.444	0.072	0.072	0.017	0.091	0.009	0.235	0.035	0.224	0.040	0.728	0.026	NA	NA	NA	NA
10	5	200	20	0.3	2	0.499	0.065	0.081	0.016	0.081	0.009	0.235	0.033	0.231	0.033	0.750	0.025	NA	NA	NA	NA
10	6	200	20	0.3	2	0.400	0.066	0.075	0.019	0.112	0.009	0.219	0.031	0.205	0.040	0.729	0.026	NA	NA	NA	NA
10	7	200	20	0.3	2	0.458	0.073	0.088	0.015	0.104	0.009	0.202	0.035	0.181	0.033	0.716	0.028	NA	NA	NA	NA
10	8	200	20	0.3	2	0.455	0.072	0.085	0.018	0.101	0.010	0.264	0.033	0.251	0.040	0.701	0.025	NA	NA	NA	NA
10	9	200	20	0.3	2	0.493	0.063	0.092	0.016	0.094	0.012	0.207	0.035	0.198	0.033	0.761	0.026	NA	NA	NA	NA
10	10	200	20	0.3	2	0.419	0.072	0.077	0.016	0.107	0.008	0.228	0.033	0.217	0.033	0.737	0.025	NA	NA	NA	NA
1	1	200	40	0.3	2	0.806	0.031	0.159	0.019	0.038	0.005	0.300	0.030	0.303	0.035	0.708	0.022	NA	NA	NA	NA
1	2	200	40	0.3	2	0.800	0.031	0.151	0.018	0.038	0.005	0.300	0.030	0.300	0.034	0.701	0.022	NA	NA	NA	NA
1	3	200	40	0.3	2	0.836	0.027	0.179	0.020	0.035	0.005	0.340	0.032	0.348	0.039	0.676	0.024	NA	NA	NA	NA
1	4	200	40	0.3	2	0.822	0.031	0.166	0.016	0.036	0.005	0.327	0.030	0.327	0.035	0.673	0.021	NA	NA	NA	NA
1	5	200	40	0.3	2	0.827	0.031	0.170	0.017	0.035	0.005	0.312	0.030	0.326	0.039	0.718	0.019	NA	NA	NA	NA
1	6	200	40	0.3	2	0.849	0.031	0.181	0.019	0.032	0.005	0.321	0.030	0.325	0.035	0.686	0.021	NA	NA	NA	NA
1	7	200	40	0.3	2	0.820	0.031	0.165	0.018	0.036	0.005	0.325	0.030	0.334	0.034	0.694	0.020	NA	NA	NA	NA
1	8	200	40	0.3	2	0.836	0.031	0.175	0.020	0.034	0.005	0.317	0.030	0.327	0.039	0.705	0.021	NA	NA	NA	NA
1	9	200	40	0.3	2	0.814	0.031	0.166	0.016	0.038	0.005	0.312	0.030	0.320	0.035	0.707	0.022	NA	NA	NA	NA
1	10	200	40	0.3	2	0.826	0.031	0.172	0.017	0.036	0.005	0.310	0.030	0.317	0.039	0.704	0.022	NA	NA	NA	NA
5	1	200	40	0.3	2	0.768	0.039	0.127	0.018	0.038	0.004	0.250	0.029	0.242	0.032	0.727	0.021	NA	NA	NA	NA
5	2	200	40	0.3	2	0.679	0.050	0.102	0.016	0.048	0.005	0.236	0.028	0.229	0.030	0.742	0.020	NA	NA	NA	NA
5	3	200	40	0.3	2	0.729	0.045	0.108	0.017	0.040	0.005	0.228	0.027	0.226	0.030	0.766	0.021	NA	NA	NA	NA
5	4	200	40	0.3	2	0.727	0.039	0.114	0.018	0.043	0.005	0.254	0.029	0.251	0.030	0.737	0.021	NA	NA	NA	NA
5	5	200	40	0.3	2	0.778	0.050	0.139	0.016	0.040	0.005	0.236	0.027	0.228	0.030	0.736	0.022	NA	NA	NA	NA
5	6	200	40	0.3	2	0.766	0.039	0.120	0.018	0.037	0.005	0.253	0.029	0.246	0.030	0.727	0.022	NA	NA	NA	NA
5	7	200	40	0.3	2	0.735	0.050	0.114	0.016	0.041	0.005	0.245	0.028	0.240	0.030	0.738	0.021	NA	NA	NA	NA
5	8	200	40	0.3	2	0.745	0.045	0.114	0.018	0.039	0.005	0.251	0.027	0.244	0.030	0.728	0.020	NA	NA	NA	NA
5	9	200	40	0.3	2	0.737	0.039	0.114	0.016	0.041	0.005	0.224	0.029	0.218	0.030	0.756	0.020	NA	NA	NA	NA
5	10	200	40	0.3	2	0.709	0.050	0.108	0.016	0.044	0.005	0.241	0.027	0.243	0.032	0.765	0.021	NA	NA	NA	NA
10	1	200	40	0.3	2	0.690	0.051	0.090	0.016	0.040	0.004	0.202	0.026	0.189	0.027	0.745	0.018	NA	NA	NA	NA
10	2	200	40	0.3	2	0.655	0.057	0.087	0.016	0.046	0.005	0.206	0.026	0.196	0.028	0.755	0.019	NA	NA	NA	NA
10	3	200	40	0.3	2	0.629	0.060	0.071	0.013	0.042	0.005	0.160	0.023	0.144	0.022	0.758	0.017	NA	NA	NA	NA
10	4	200	40	0.3	2	0.642	0.051	0.079	0.016	0.044	0.005	0.188	0.023	0.173	0.027	0.750	0.019	NA	NA	NA	NA
10	5	200	40	0.3	2	0.662	0.057	0.081	0.016	0.041	0.005	0.184	0.026	0.166	0.028	0.739	0.017	NA	NA	NA	NA
10	6	200	40	0.3	2	0.650	0.051	0.081	0.016	0.044	0.005	0.175	0.026	0.160	0.027	0.757	0.018	NA	NA	NA	NA
10	7	200	40	0.3	2	0.651	0.057	0.081	0.016	0.043	0.005	0.172	0.026	0.157	0.028	0.759	0.019	NA	NA	NA	NA
10	8	200	40	0.3	2	0.644	0.060	0.079	0.016	0.044	0.005	0.174	0.026	0.158	0.027	0.749	0.017	NA	NA	NA	NA
10	9	200	40	0.3	2	0.663	0.051	0.079	0.016	0.040	0.005	0.194	0.026	0.182	0.028	0.756	0.019	NA	NA	NA	NA
10	10	200	40	0.3	2	0.642	0.057	0.080	0.016	0.045	0.005	0.169	0.026	0.151	0.022	0.745	0.017	NA	NA	NA	NA
1	1	200	60	0.3	2	0.865	0.022	0.167	0.015	0.026	0.004	0.335	0.031	0.341	0.037	0.679	0.022	0.843	0.020	0.167	0.889
1	2	200	60	0.3	2	0.899	0.018	0.159	0.017	0.018	0.004	0.353	0.032	0.362	0.039	0.664	0.023	0.860	0.018	0.176	0.896
1	3	200	60	0.3	2	0.850	0.024	0.187	0.018	0.033	0.005	0.316	0.029	0.313	0.034	0.679	0.020	0.828	0.022	0.158	0.877
1	4	200	60	0.3	2	0.867	0.022	0.174	0.015	0.027	0.004	0.312	0.031	0.349	0.037	0.770	0.022	0.856	0.020	0.150	0.904
1	5	200	60	0.3	2	0.848	0.024	0.178	0.017	0.032	0.004	0.303	0.029	0.335	0.034	0.772	0.020	0.842	0.022	0.160	0.833
1	6	200	60	0.3	2	0.863	0.022	0.189	0.017	0.030	0.004	0.302	0.032	0.334	0.037	0.771	0.022	0.837	0.020	0.117	1.066
1	7	200	60	0.3	2	0.856	0.018	0.173	0.017	0.029	0.004	0.300	0.029	0.331	0.034	0.771	0.023	0.841	0.018	0.178	0.755
1	8	200	60	0.3	2	0.868	0.024	0.183	0.017	0.028	0.005	0.296	0.031	0.325	0.039	0.771	0.020	0.860	0.022	0.150	0.858
1	9	200	60	0.3	2	0.892	0.022	0.174	0.015	0.021	0.004	0.312	0.032	0.349	0.037	0.770	0.022	0.852	0.020	0.175	0.796
1	10	200	60	0.3	2	0.882	0.018	0.180	0.017	0.024	0.004	0.315	0.029	0.354	0.034	0.771	0.020	0.844	0.022	0.157	0.879
5	1	200	60	0.3	2	0.670	0.056	0.135	0.012	0.066	0.003	0.203	0.024	0.185	0.025	0.725	0.014	0.648	0.041	0.057	1.186
5	2	200	60	0.3	2	0.762	0.041	0.110	0.017	0.034	0.004	0.188	0.022	0.174	0.023	0.749	0.015	0.676	0.038	0.063	1.031
5	3	200	60	0.3	2	0.772	0.038	0.116	0.012	0.034	0.005	0.229	0.025	0.217	0.027	0.728	0.017	0.685	0.038	0.076	1.108
5	4	200	60	0.3	2	0.677	0.056	0.122	0.012	0.058	0.004	0.198	0.025	0.182	0.027	0.737	0.014	0.683	0.038	0.067	1.043
5	5	200	60	0.3	2	0.700	0.038	0.147	0.012	0.063	0.004	0.200	0.026	0.184	0.026	0.737	0.016	0.680	0.038	0.066	1.064
5	6	200	60	0.3	2	0.683	0.041	0.128	0.012	0.059	0.004	0.226	0.024	0.215	0.025	0.736	0.016	0.666	0.038	0.065	1.214
5	7	200	60	0.3	2	0.741	0.038	0.122	0.012	0.043	0.004	0.206	0.022	0.191	0.023	0.737	0.014	0.655	0.038	0.071	1.044
5	8	200	60	0.3	2	0.752	0.056	0.122	0.012	0.040	0.004	0.211	0.025	0.197	0.027	0.737	0.015	0.667	0.038	0.064	1.145
5	9	200	60	0.3	2	0.710	0.041	0.122	0.012	0.050	0.004	0.233	0.025	0.225	0.027	0.739	0.015	0.655	0.038	0.067	1.227
5	10	200	60	0.3	2	0.718	0.038	0.116	0.012	0.046	0.004	0.205	0.026	0.190	0.026	0.737	0.017	0.677	0.038	0.068	1.069
10	1	200	60	0.3	2	0.700	0.053	0.098	0.008	0.042	0.004	0.217	0.025	0.201	0.027	0.726	0.014	0.638	0.042	0.055	1.296
10	2	200	60	0.3	2	0.794	0.036	0.095	0.006	0.025	0.005	0.242	0.026	0.229	0.029	0.715	0.016	0.680	0.038	0.069	1.250
10	3	200	60	0.3	2	0.825	0.032	0.079	0.002	0.017	0.005	0.214	0.024	0.202	0.026	0.740	0.016	0.706	0.035	0.059	1.224
10	4	200	60	0.3	2	0.797	0.038	0.087	0.006	0.022	0.005	0.234	0.024	0.218	0.025	0.716	0.014	0.682	0.041	0.063	1.283
10	5	200	60	0.3	2	0.739	0.056	0.089	0.002	0.031	0.005	0.227	0.022	0.217	0.023	0.737	0.015	0.679	0.038	0.058	1.312
10	6	200	60	0.3	2	0.758	0.041	0.089	0.008	0.028	0.005	0.226	0.025	0.212	0.027	0.730	0.014	0.682	0.038	0.059	1.292
10	7	200	60	0.3	2	0.759	0.038	0.089	0.008	0.028	0.005	0.228	0.022	0.215	0.027	0.728	0.016	0.702	0.042	0.060	1.290
10	8	200	60	0.3	2	0.729	0.053	0.087	0.008	0.032	0.005	0.234	0.025	0.225	0.026	0.737	0.016	0.675	0.038	0.061	1.310
10	9	200	60	0.3	2	0.770	0.036	0.087	0.006	0.026	0.005	0.228	0.025	0.214	0.029	0.725	0.014	0.658	0.035	0.064	1.237
10	10	200	60	0.3	2	0.765	0.032	0.088	0.002	0.027	0.005	0.225	0.026	0.208	0.026	0.717	0.015	0.666	0.041	0.059	1.286
1	1	100	20	0.3	5	0.427	0.059	0.154	0.029	0.207	0.019	0.313	0.049	0.323	0.058	0.706	0.041	NA	NA	NA	NA
1	2	100	20	0.3	5	0.422	0.058	0.137	0.025	0.188	0.017	0.280	0.046	0.286	0.053	0.734	0.040	NA	NA	NA	NA
1	3	100	20	0.3	5	0.383	0.057	0.120	0.023	0.193	0.017	0.273	0.045	0.258	0.048	0.687	0.037	NA	NA	NA	NA
1	4	100	20	0.3	5	0.431	0.058	0.140	0.029	0.185	0.017	0.293	0.046	0.303	0.056	0.732	0.042	NA	NA	NA	NA
1	5	100	20	0.3	5	0.435	0.057	0.149	0.023	0.193	0.017	0.271	0.045	0.273	0.052	0.736	0.035	NA	NA	NA	NA
1	6	100	20	0.3	5	0.428	0.059	0.153	0.029	0.205	0.019	0.241	0.043	0.236	0.046	0.741	0.038	NA	NA	NA	NA
1	7	100	20	0.3	5	0.419	0.058	0.150	0.025	0.208	0.017	0.281	0.046	0.291	0.051	0.744	0.039	NA	NA	NA	NA
1	8	100	20	0.3	5	0.414	0.057	0.143	0.023	0.203	0.017	0.286	0.045	0.284	0.053	0.710	0.041	NA	NA	NA	NA
1	9	100	20	0.3	5	0.401	0.058	0.132	0.029	0.197	0.017	0.293	0.048	0.310	0.056	0.749	0.036	NA	NA	NA	NA
1	10	100	20	0.3	5	0.383	0.057	0.129	0.023	0.207	0.017	0.287	0.045	0.294	0.050	0.731	0.040	NA	NA	NA	NA
5	1	100	20	0.3	5	0.376	0.066	0.123	0.028	0.204	0.018	0.263	0.052	0.275	0.062	0.769	0.043	NA	NA	NA	NA
5	2	100	20	0.3	5	0.395	0.061	0.116	0.024	0.178	0.015	0.252	0.049	0.243	0.054	0.721	0.039	NA	NA	NA	NA
5	3	100	20	0.3	5	0.240	0.060	0.070	0.020	0.222	0.018	0.156	0.040	0.141	0.038	0.765	0.034	NA	NA	NA	NA
5	4	100	20	0.3	5	0.377	0.066	0.114	0.028	0.188	0.018	0.258	0.050	0.264	0.062	0.761	0.042	NA	NA	NA	NA
5	5	100	20	0.3	5	0.363	0.060	0.093	0.020	0.162	0.018	0.228	0.047	0.217	0.055	0.733	0.039	NA	NA	NA	NA
5	6	100	20	0.3	5	0.274	0.066	0.074	0.028	0.196	0.018	0.246	0.042	0.250	0.037	0.763	0.035	NA	NA	NA	NA
5	7	100	20	0.3	5	0.350	0.061	0.088	0.024	0.164	0.015	0.266	0.051	0.272	0.028	0.750	0.031	NA	NA	NA	NA
5	8	100	20	0.3	5	0.298	0.060	0.100	0.020	0.236	0.018	0.242	0.049	0.238	0.064	0.747	0.039	NA	NA	NA	NA
5	9	100	20	0.3	5	0.287	0.066	0.090	0.028	0.224	0.018	0.238	0.045	0.235	0.052	0.753	0.038	NA	NA	NA	NA
5	10	100	20	0.3	5	0.305	0.060	0.099	0.020	0.226	0.018	0.237	0.052	0.238	0.035	0.766	0.033	NA	NA	NA	NA
10	1	100	20	0.3	5	0.224	0.057	0.063	0.018	0.220	0.017	0.225	0.049	0.217	0.053	0.748	0.038	NA	NA	NA	NA
10	2	100	20	0.3	5	0.323	0.060	0.091	0.021	0.191	0.015	0.253	0.051	0.240	0.055	0.710	0.037	NA	NA	NA	NA
10	3	100	20	0.3	5	0.483	0.059	0.155	0.029	0.166	0.014	0.352	0.058	0.356	0.071	0.655	0.042	NA	NA	NA	NA
10	4	100	20	0.3	5	0.297	0.060	0.087	0.018	0.206	0.017	0.294	0.043	0.289	0.056	0.695	0.033	NA	NA	NA	NA
10	5	100	20	0.3	5	0.367	0.059	0.101	0.029	0.174	0.014	0.284	0.045	0.285	0.058	0.718	0.035	NA	NA	NA	NA
10	6	100	20	0.3	5	0.219	0.057	0.054	0.018	0.194	0.017	0.303	0.047	0.327	0.060	0.754	0.037	NA	NA	NA	NA
10	7	100	20	0.3	5	0.476	0.060	0.130	0.021	0.143	0.015	0.256	0.049	0.254	0.062	0.738	0.039	NA	NA	NA	NA
10	8	100	20	0.3	5	0.268	0.059	0.059	0.029	0.162	0.014	0.282	0.051	0.289	0.064	0.737	0.041	NA	NA	NA	NA
10	9	100	20	0.3	5	0.537	0.060	0.184	0.018	0.158	0.017	0.310	0.053	0.323	0.066	0.721	0.043	NA	NA	NA	NA
10	10	100	20	0.3	5	0.370	0.059	0.098	0.029	0.167	0.014	0.261	0.055	0.246	0.068	0.695	0.045	NA	NA	NA	NA
1	1	100	40	0.3	5	0.572	0.052	0.129	0.021	0.097	0.008	0.263	0.038	0.261	0.043	0.731	0.029	NA	NA	NA	NA
1	2	100	40	0.3	5	0.642	0.050	0.142	0.023	0.079	0.007	0.281	0.040	0.263	0.043	0.673	0.029	NA	NA	NA	NA
1	3	100	40	0.3	5	0.633	0.050	0.167	0.027	0.097	0.009	0.316	0.043	0.321	0.052	0.693	0.033	NA	NA	NA	NA
1	4	100	40	0.3	5	0.553	0.050	0.129	0.023	0.104	0.007	0.277	0.038	0.279	0.043	0.730	0.029	NA	NA	NA	NA
1	5	100	40	0.3	5	0.643	0.050	0.142	0.027	0.079	0.009	0.296	0.040	0.291	0.052	0.693	0.033	NA	NA	NA	NA
1	6	100	40	0.3	5	0.648	0.050	0.169	0.021	0.092	0.008	0.300	0.038	0.313	0.043	0.729	0.029	NA	NA	NA	NA
1	7	100	40	0.3	5	0.584	0.050	0.129	0.023	0.092	0.007	0.290	0.040	0.291	0.043	0.713	0.029	NA	NA	NA	NA
1	8	100	40	0.3	5	0.574	0.050	0.141	0.027	0.104	0.009	0.283	0.043	0.278	0.052	0.703	0.033	NA	NA	NA	NA
1	9	100	40	0.3	5	0.680	0.050	0.168	0.023	0.079	0.007	0.296	0.038	0.309	0.043	0.734	0.029	NA	NA	NA	NA
1	10	100	40	0.3	5	0.591	0.050	0.133	0.027	0.092	0.009	0.307	0.040	0.301	0.052	0.679	0.033	NA	NA	NA	NA
5	1	100	40	0.3	5	0.402	0.060	0.072	0.015	0.107	0.008	0.202	0.036	0.188	0.037	0.741	0.026	NA	NA	NA	NA
5	2	100	40	0.3	5	0.579	0.056	0.142	0.027	0.103	0.008	0.300	0.045	0.308	0.055	0.720	0.032	NA	NA	NA	NA
5	3	100	40	0.3	5	0.430	0.062	0.067	0.014	0.088	0.007	0.162	0.032	0.143	0.030	0.737	0.023	NA	NA	NA	NA
5	4	100	40	0.3	5	0.444	0.060	0.085	0.027	0.106	0.008	0.196	0.036	0.178	0.037	0.730	0.026	NA	NA	NA	NA
5	5	100	40	0.3	5	0.515	0.056	0.103	0.014	0.097	0.007	0.234	0.032	0.228	0.030	0.746	0.023	NA	NA	NA	NA
5	6	100	40	0.3	5	0.499	0.060	0.099	0.015	0.100	0.008	0.199	0.036	0.182	0.037	0.732	0.026	NA	NA	NA	NA
5	7	100	40	0.3	5	0.531	0.056	0.097	0.027	0.085	0.008	0.195	0.045	0.177	0.055	0.729	0.032	NA	NA	NA	NA
5	8	100	40	0.3	5	0.502	0.060	0.094	0.014	0.094	0.007	0.223	0.032	0.210	0.030	0.732	0.023	NA	NA	NA	NA
5	9	100	40	0.3	5	0.500	0.056	0.102	0.027	0.102	0.008	0.245	0.036	0.232	0.037	0.715	0.026	NA	NA	NA	NA
5	10	100	40	0.3	5	0.444	0.056	0.088	0.014	0.111	0.007	0.227	0.032	0.217	0.030	0.738	0.023	NA	NA	NA	NA

10	1	100	40	0.3	5	0.379	0.064	0.063	0.015	0.103	0.008	0.201	0.037	0.185	0.039	0.735	0.024	NA	NA	NA	NA
10	2	100	40	0.3	5	0.383	0.063	0.062	0.015	0.100	0.007	0.158	0.032	0.147	0.033	0.781	0.023	NA	NA	NA	NA
10	3	100	40	0.3	5	0.441	0.062	0.072	0.016	0.092	0.007	0.205	0.037	0.184	0.037	0.714	0.024	NA	NA	NA	NA
10	4	100	40	0.3	5	0.405	0.064	0.058	0.015	0.085	0.007	0.138	0.037	0.128	0.039	0.798	0.024	NA	NA	NA	NA
10	5	100	40	0.3	5	0.404	0.063	0.067	0.015	0.099	0.007	0.185	0.037	0.165	0.037	0.730	0.023	NA	NA	NA	NA
10	6	100	40	0.3	5	0.458	0.064	0.072	0.015	0.085	0.008	0.193	0.037	0.177	0.039	0.742	0.024	NA	NA	NA	NA
10	7	100	40	0.3	5	0.369	0.063	0.058	0.015	0.099	0.007	0.215	0.032	0.209	0.033	0.762	0.023	NA	NA	NA	NA
10	8	100	40	0.3	5	0.419	0.062	0.067	0.016	0.093	0.007	0.224	0.037	0.216	0.037	0.749	0.024	NA	NA	NA	NA
10	9	100	40	0.3	5	0.444	0.064	0.066	0.015	0.083	0.007	0.174	0.037	0.150	0.039	0.716	0.024	NA	NA	NA	NA
10	10	100	40	0.3	5	0.399	0.063	0.067	0.015	0.101	0.007	0.223	0.037	0.212	0.037	0.740	0.023	NA	NA	NA	NA
1	1	100	60	0.3	5	0.729	0.040	0.143	0.022	0.053	0.005	0.290	0.039	0.289	0.045	0.707	0.028	NA	NA	NA	NA
1	2	100	60	0.3	5	0.765	0.037	0.155	0.023	0.048	0.005	0.311	0.041	0.311	0.048	0.690	0.029	NA	NA	NA	NA
1	3	100	60	0.3	5	0.728	0.040	0.160	0.024	0.060	0.005	0.328	0.043	0.339	0.052	0.695	0.031	NA	NA	NA	NA
1	4	100	60	0.3	5	0.713	0.040	0.134	0.022	0.054	0.005	0.295	0.039	0.292	0.045	0.698	0.028	NA	NA	NA	NA
1	5	100	60	0.3	5	0.749	0.040	0.146	0.022	0.049	0.005	0.316	0.041	0.314	0.052	0.681	0.029	NA	NA	NA	NA
1	6	100	60	0.3	5	0.712	0.040	0.151	0.022	0.061	0.005	0.333	0.039	0.342	0.045	0.686	0.028	NA	NA	NA	NA
1	7	100	60	0.3	5	0.738	0.040	0.144	0.022	0.051	0.005	0.286	0.041	0.286	0.048	0.713	0.029	NA	NA	NA	NA
1	8	100	60	0.3	5	0.772	0.040	0.156	0.022	0.046	0.005	0.307	0.043	0.308	0.052	0.696	0.031	NA	NA	NA	NA
1	9	100	60	0.3	5	0.735	0.040	0.161	0.022	0.058	0.005	0.324	0.039	0.336	0.045	0.701	0.028	NA	NA	NA	NA
1	10	100	60	0.3	5	0.722	0.040	0.135	0.022	0.052	0.005	0.291	0.041	0.289	0.052	0.704	0.029	NA	NA	NA	NA
5	1	100	60	0.3	5	0.589	0.054	0.104	0.019	0.072	0.006	0.223	0.035	0.214	0.038	0.743	0.023	NA	NA	NA	NA
5	2	100	60	0.3	5	0.658	0.050	0.122	0.022	0.063	0.005	0.255	0.038	0.257	0.044	0.752	0.025	NA	NA	NA	NA
5	3	100	60	0.3	5	0.585	0.054	0.098	0.018	0.070	0.005	0.217	0.034	0.200	0.035	0.724	0.022	NA	NA	NA	NA
5	4	100	60	0.3	5	0.597	0.054	0.105	0.022	0.071	0.005	0.229	0.038	0.218	0.038	0.734	0.023	NA	NA	NA	NA
5	5	100	60	0.3	5	0.665	0.054	0.123	0.022	0.062	0.005	0.260	0.034	0.261	0.035	0.743	0.022	NA	NA	NA	NA
5	6	100	60	0.3	5	0.589	0.054	0.099	0.022	0.069	0.005	0.222	0.035	0.204	0.038	0.715	0.023	NA	NA	NA	NA
5	7	100	60	0.3	5	0.581	0.054	0.102	0.022	0.074	0.005	0.220	0.038	0.211	0.044	0.748	0.025	NA	NA	NA	NA
5	8	100	60	0.3	5	0.650	0.054	0.120	0.022	0.065	0.005	0.251	0.034	0.254	0.035	0.757	0.022	NA	NA	NA	NA
5	9	100	60	0.3	5	0.573	0.054	0.096	0.022	0.072	0.005	0.213	0.038	0.197	0.038	0.729	0.023	NA	NA	NA	NA
5	10	100	60	0.3	5	0.587	0.054	0.103	0.022	0.073	0.005	0.225	0.034	0.215	0.035	0.739	0.022	NA	NA	NA	NA
10	1	100	60	0.3	5	0.548	0.058	0.076	0.015	0.062	0.005	0.179	0.031	0.167	0.033	0.763	0.020	NA	NA	NA	NA
10	2	100	60	0.3	5	0.491	0.066	0.067	0.016	0.070	0.005	0.178	0.032	0.161	0.032	0.741	0.019	NA	NA	NA	NA
10	3	100	60	0.3	5	0.295	0.069	0.032	0.010	0.077	0.006	0.153	0.030	0.131	0.028	0.726	0.017	NA	NA	NA	NA
10	4	100	60	0.3	5	0.551	0.069	0.075	0.016	0.061	0.005	0.184	0.035	0.170	0.032	0.754	0.020	NA	NA	NA	NA
10	5	100	60	0.3	5	0.489	0.066	0.066	0.016	0.069	0.005	0.183	0.038	0.164	0.028	0.732	0.017	NA	NA	NA	NA
10	6	100	60	0.3	5	0.290	0.066	0.031	0.016	0.076	0.005	0.157	0.034	0.134	0.033	0.717	0.020	NA	NA	NA	NA
10	7	100	60	0.3	5	0.562	0.058	0.078	0.016	0.061	0.005	0.177	0.038	0.165	0.032	0.767	0.019	NA	NA	NA	NA
10	8	100	60	0.3	5	0.500	0.058	0.069	0.016	0.069	0.005	0.176	0.034	0.159	0.028	0.745	0.017	NA	NA	NA	NA
10	9	100	60	0.3	5	0.307	0.058	0.034	0.010	0.076	0.005	0.150	0.031	0.129	0.032	0.730	0.020	NA	NA	NA	NA
10	10	100	60	0.3	5	0.563	0.058	0.077	0.010	0.060	0.005	0.181	0.030	0.168	0.028	0.758	0.017	NA	NA	NA	NA
1	1	200	20	0.3	5	0.379	0.040	0.115	0.016	0.189	0.012	0.247	0.030	0.244	0.033	0.741	0.027	NA	NA	NA	NA
1	2	200	20	0.3	5	0.475	0.041	0.172	0.021	0.190	0.013	0.348	0.037	0.359	0.045	0.673	0.030	NA	NA	NA	NA
1	3	200	20	0.3	5	0.450	0.041	0.143	0.018	0.174	0.011	0.298	0.034	0.285	0.037	0.669	0.027	NA	NA	NA	NA
1	4	200	20	0.3	5	0.399	0.041	0.122	0.016	0.183	0.011	0.323	0.030	0.326	0.033	0.684	0.030	NA	NA	NA	NA
1	5	200	20	0.3	5	0.451	0.041	0.144	0.017	0.176	0.011	0.293	0.034	0.298	0.037	0.717	0.027	NA	NA	NA	NA
1	6	200	20	0.3	5	0.513	0.041	0.181	0.015	0.172	0.011	0.311	0.030	0.314	0.033	0.695	0.027	NA	NA	NA	NA
1	7	200	20	0.3	5	0.499	0.041	0.169	0.013	0.170	0.011	0.275	0.037	0.267	0.045	0.703	0.027	NA	NA	NA	NA
1	8	200	20	0.3	5	0.495	0.041	0.178	0.016	0.182	0.012	0.293	0.034	0.307	0.037	0.742	0.027	NA	NA	NA	NA
1	9	200	20	0.3	5	0.419	0.041	0.126	0.017	0.175	0.013	0.286	0.030	0.285	0.033	0.713	0.027	NA	NA	NA	NA
1	10	200	20	0.3	5	0.505	0.041	0.183	0.015	0.179	0.011	0.291	0.034	0.291	0.037	0.709	0.030	NA	NA	NA	NA
5	1	200	20	0.3	5	0.252	0.042	0.068	0.013	0.201	0.011	0.183	0.030	0.165	0.030	0.737	0.025	NA	NA	NA	NA
5	2	200	20	0.3	5	0.352	0.044	0.105	0.017	0.193	0.011	0.232	0.034	0.217	0.035	0.719	0.026	NA	NA	NA	NA
5	3	200	20	0.3	5	0.268	0.041	0.070	0.013	0.190	0.011	0.166	0.029	0.151	0.028	0.761	0.025	NA	NA	NA	NA
5	4	200	20	0.3	5	0.281	0.041	0.078	0.016	0.198	0.011	0.212	0.030	0.197	0.030	0.734	0.025	NA	NA	NA	NA
5	5	200	20	0.3	5	0.332	0.041	0.092	0.017	0.186	0.011	0.163	0.029	0.141	0.035	0.724	0.026	NA	NA	NA	NA
5	6	200	20	0.3	5	0.239	0.041	0.066	0.015	0.211	0.011	0.210	0.030	0.190	0.030	0.715	0.025	NA	NA	NA	NA
5	7	200	20	0.3	5	0.334	0.041	0.095	0.013	0.189	0.011	0.225	0.034	0.207	0.035	0.715	0.026	NA	NA	NA	NA
5	8	200	20	0.3	5	0.243	0.041	0.064	0.016	0.199	0.011	0.219	0.029	0.202	0.028	0.723	0.025	NA	NA	NA	NA
5	9	200	20	0.3	5	0.373	0.041	0.115	0.017	0.194	0.011	0.209	0.030	0.196	0.030	0.742	0.025	NA	NA	NA	NA
5	10	200	20	0.3	5	0.305	0.041	0.081	0.015	0.186	0.011	0.225	0.029	0.209	0.035	0.721	0.026	NA	NA	NA	NA
10	1	200	20	0.3	5	0.294	0.043	0.087	0.016	0.209	0.012	0.199	0.031	0.188	0.032	0.755	0.026	NA	NA	NA	NA
10	2	200	20	0.3	5	0.337	0.044	0.104	0.017	0.204	0.011	0.247	0.035	0.248	0.040	0.757	0.028	NA	NA	NA	NA
10	3	200	20	0.3	5	0.350	0.042	0.093	0.015	0.174	0.010	0.224	0.033	0.202	0.033	0.699	0.025	NA	NA	NA	NA
10	4	200	20	0.3	5	0.245	0.041	0.065	0.016	0.200	0.011	0.235	0.035	0.224	0.040	0.728	0.026	NA	NA	NA	NA
10	5	200	20	0.3	5	0.346	0.041	0.096	0.021	0.181	0.011	0.235	0.033	0.231	0.033	0.750	0.025	NA	NA	NA	NA
10	6	200	20	0.3	5	0.343	0.041	0.102	0.018	0.195	0.011	0.219	0.031	0.205	0.040	0.729	0.026	NA	NA	NA	NA
10	7	200	20	0.3	5	0.347	0.044	0.095	0.013	0.178	0.011	0.202	0.035	0.181	0.033	0.716	0.028	NA	NA	NA	NA
10	8	200	20	0.3	5	0.352	0.042	0.104	0.016	0.191	0.011	0.264	0.033	0.251	0.040	0.701	0.025	NA	NA	NA	NA
10	9	200	20	0.3	5	0.356	0.041	0.108	0.017	0.196	0.011	0.207	0.035	0.198	0.033	0.761	0.026	NA	NA	NA	NA
10	10	200	20	0.3	5	0.364	0.041	0.115	0.015	0.201	0.011	0.228	0.033	0.217	0.033	0.737	0.025	NA	NA	NA	NA
1	1	200	40	0.3	5	0.625	0.034	0.153	0.017	0.092	0.006	0.300	0.030	0.303	0.035	0.708	0.022	NA	NA	NA	NA
1	2	200	40	0.3	5	0.644	0.035	0.155	0.018	0.086	0.006	0.300	0.030	0.300	0.034	0.701	0.022	NA	NA	NA	NA
1	3	200	40	0.3	5	0.664	0.033	0.169	0.018	0.086	0.006	0.340	0.032	0.348	0.039	0.676	0.024	NA	NA	NA	NA
1	4	200	40	0.3	5	0.645	0.034	0.160	0.018	0.088	0.006	0.327	0.030	0.327	0.035	0.673	0.021	NA	NA	NA	NA
1	5	200	40	0.3	5	0.642	0.033	0.160	0.018	0.089	0.006	0.312	0.030	0.326	0.039	0.718	0.019	NA	NA	NA	NA
1	6	200	40	0.3	5	0.648	0.034	0.162	0.018	0.088	0.006	0.321	0.030	0.325	0.035	0.686	0.021	NA	NA	NA	NA
1	7	200	40	0.3	5	0.641	0.035	0.160	0.018	0.090	0.006	0.325	0.030	0.334	0.034	0.694	0.020	NA	NA	NA	NA
1	8	200	40	0.3	5	0.648	0.033	0.161	0.018	0.088	0.006	0.317	0.030	0.327	0.039	0.705	0.021	NA	NA	NA	NA
1	9	200	40	0.3	5	0.640	0.034	0.160	0.018	0.090	0.006	0.312	0.030	0.320	0.035	0.707	0.022	NA	NA	NA	NA
1	10	200	40	0.3	5	0.645	0.033	0.161	0.018	0.089	0.006	0.310	0.030	0.317	0.039	0.704	0.022	NA	NA	NA	NA
5	1	200	40	0.3	5	0.541	0.041	0.116	0.016	0.099	0.006	0.250	0.029	0.242	0.032	0.727	0.021	NA	NA	NA	NA
5	2	200	40	0.3	5	0.540	0.040	0.114	0.015	0.097	0.006	0.236	0.028	0.229	0.030	0.742	0.020	NA	NA	NA	NA
5	3	200	40	0.3	5	0.522	0.042	0.107	0.015	0.098	0.006	0.228	0.027	0.226	0.030	0.766	0.021	NA	NA	NA	NA
5	4	200	40	0.3	5	0.531	0.041	0.112	0.015	0.099	0.006	0.254	0.029	0.251	0.030	0.737	0.021	NA	NA	NA	NA
5	5	200	40	0.3	5	0.526	0.042	0.110	0.015	0.099	0.006	0.236	0.027	0.228	0.030	0.736	0.022	NA	NA	NA	NA
5	6	200	40	0.3	5	0.535	0.041	0.113	0.015	0.098	0.006	0.253	0.029	0.246	0.030	0.727	0.022	NA	NA	NA	NA
5	7	200	40	0.3	5	0.534	0.040	0.111	0.015	0.097	0.006	0.245	0.028	0.240	0.030	0.738	0.021	NA	NA	NA	NA
5	8	200	40	0.3	5	0.534	0.042	0.111	0.015	0.097	0.006	0.251	0.027	0.244	0.030	0.728	0.020	NA	NA	NA	NA
5	9	200	40	0.3	5	0.534	0.041	0.112	0.015	0.097	0.006	0.224	0.029	0.218	0.030	0.756	0.020	NA	NA	NA	NA
5	10	200	40	0.3	5	0.535	0.042	0.111	0.015	0.097	0.006	0.241	0.027	0.243	0.032	0.765	0.021	NA	NA	NA	NA
10	1	200	40	0.3	5	0.433	0.046	0.079	0.013	0.103	0.006	0.202	0.026	0.189	0.027	0.745	0.018	NA	NA	NA	NA
10	2	200	40	0.3	5	0.481	0.045	0.095	0.015	0.103	0.006	0.206	0.026	0.196	0.028	0.755	0.019	NA	NA	NA	NA
10	3	200	40	0.3	5	0.460	0.046	0.082	0.013	0.096	0.005	0.160	0.023	0.144	0.022	0.758	0.017	NA	NA	NA	NA
10	4	200	40	0.3	5	0.461	0.046	0.087	0.013	0.101	0.006	0.188	0.023	0.173	0.027	0.750	0.019	NA	NA	NA	NA
10	5	200	40	0.3	5	0.471	0.046	0.087	0.013	0.098	0.006	0.184	0.026	0.166	0.028	0.739	0.017	NA	NA	NA	NA
10	6	200	40	0.3	5	0.461	0.046	0.086	0.013	0.100	0.006	0.175	0.026	0.160	0.027	0.757	0.018	NA	NA	NA	NA
10	7	200	40	0.3	5	0.461	0.046	0.086	0.013	0.100	0.006	0.172	0.026	0.157	0.028	0.759	0.019	NA	NA	NA	NA
10	8	200	40	0.3	5	0.460	0.046	0.086	0.013	0.101	0.006	0.174	0.026	0.158	0.027	0.749	0.017	NA	NA	NA	NA
10	9	200	40	0.3	5	0.471	0.046	0.088	0.013	0.099	0.006	0.194	0.026	0.182	0.028	0.756	0.019	NA	NA	NA	NA
10	10	200	40	0.3	5	0.468	0.046	0.088	0.013	0.100	0.006	0.169	0.026	0.151	0.022	0.745	0.017	NA	NA	NA	NA
1	1	200	60	0.3	5	0.763	0.026	0.145	0.016	0.045	0.003	0.335	0.031	0.341	0.037	0.679	0.022	0.721	0.024	NA	NA
1	2	200	60	0.3	5	0.764	0.026	0.155	0.018	0.048	0.003	0.353	0.032	0.362	0.039	0.664	0.023	0.725	0.024	NA	NA
1	3	200	60	0.3	5	0.718	0.028	0.144	0.019	0.056	0.004	0.316	0.029	0.313	0.034	0.679	0.020	0.688	0.026	NA	NA
1	4	200	60	0.3	5	0.739	0.026	0.173	0.016	0.061	0.003	0.312	0.031	0.349	0.037	0.770	0.022	0.714	0.024	NA	NA
1	5	200	60	0.3	5	0.713	0.026	0.175	0.018	0.071	0.003	0.303	0.029	0.335	0.034	0.772	0.020	0.699	0.024	NA	NA
1	6	200	60	0.3	5	0.727	0.026	0.150	0.018	0.056	0.003	0.302	0.032	0.334	0.037	0.771	0.022	0.734	0.024	NA	NA
1	7	200	60	0.3	5	0.712	0.026	0.165	0.018	0.067	0.003	0.300	0.029	0.331	0.034	0.771	0.023	0.743	0.024	NA	NA
1	8	200	60	0.3	5	0.732	0.026	0.171	0.018	0.063	0.004	0.296	0.031	0.325	0.039	0.771	0.020	0.715	0.024	NA	NA
1	9	200	60	0.3	5	0.752	0.026	0.164	0.016	0.054	0.003	0.312	0.032	0.349	0.037	0.770	0.022	0.701	0.024	NA	NA
1	10	200	60	0.3	5	0.732	0.026	0.169	0.018	0.062	0.003	0.315	0.029	0.354	0.034	0.771	0.020	0.695	0.024	NA	NA
5	1	200	60	0.3	5	0.555	0.046	0.111	0.013	0.089	0.002	0.203	0.024	0.185	0.025	0.725	0.014	0.400	0.035	NA	NA
5	2	200	60	0.3	5	0.527	0.043	0.121	0.018	0.109	0.003	0.188	0.022	0.174	0.023	0.749	0.015	0.420	0.035	NA	NA
5	3	200	60	0.3	5	0.675	0.034	0.111	0.013	0.053	0.004	0.229	0.025	0.217	0.027	0.728	0.017	0.501	0.034	NA	NA
5	4	200	60	0.3	5	0.596	0.046	0.126	0.013	0.085	0.003	0.198	0.025	0.182	0.027	0.737	0.014	0.430	0.035	NA	NA
5	5	200	60	0.3	5	0.542	0.036	0.104	0.013	0.088	0.003	0.200	0.026	0.184	0.026	0.737	0.016	0.457	0.035	NA	NA
5	6	200	60	0.3	5	0.556	0.040	0.131	0.013	0.105	0.003	0.226	0.024	0.215	0.025	0.736	0.016	0.464	0.035	NA	NA
5	7	200	60	0.3	5	0.563	0.043	0.119	0.013	0.092	0.003	0.206	0.022	0.191	0.023	0.737	0.014	0.467	0.035	NA	NA
5	8	200	60	0.3	5	0.573	0.034	0.116	0.013	0.086	0.003	0.211	0.025	0.197	0.027	0.737	0.015	0.419	0.035	NA	NA
5	9	200	60	0.3	5	0.558	0.046	0.122	0.013	0.096	0.003	0.233	0.025	0.225	0.027	0.739	0.015	0.498	0.035	NA	NA

5	10	200	60	0.3	5	0.554	0.036	0.117	0.013	0.095	0.003	0.205	0.026	0.190	0.026	0.737	0.017	0.425	0.035	NA	NA
10	1	200	60	0.3	5	0.535	0.046	0.082	0.009	0.072	0.003	0.217	0.025	0.201	0.027	0.726	0.014	0.412	0.035	NA	NA
10	2	200	60	0.3	5	0.665	0.036	0.087	0.007	0.044	0.004	0.242	0.026	0.229	0.029	0.715	0.016	0.482	0.034	NA	NA
10	3	200	60	0.3	5	0.610	0.040	0.076	0.003	0.048	0.004	0.214	0.024	0.202	0.026	0.740	0.016	0.408	0.035	NA	NA
10	4	200	60	0.3	5	0.651	0.046	0.082	0.007	0.044	0.004	0.234	0.024	0.218	0.025	0.716	0.014	0.472	0.035	NA	NA
10	5	200	60	0.3	5	0.621	0.043	0.083	0.003	0.050	0.004	0.227	0.022	0.217	0.023	0.737	0.015	0.473	0.035	NA	NA
10	6	200	60	0.3	5	0.536	0.034	0.089	0.009	0.077	0.004	0.226	0.025	0.212	0.027	0.730	0.014	0.414	0.035	NA	NA
10	7	200	60	0.3	5	0.648	0.036	0.098	0.009	0.053	0.004	0.228	0.022	0.215	0.027	0.728	0.016	0.453	0.035	NA	NA
10	8	200	60	0.3	5	0.544	0.040	0.086	0.009	0.072	0.004	0.234	0.025	0.225	0.026	0.737	0.016	0.440	0.035	NA	NA
10	9	200	60	0.3	5	0.536	0.046	0.098	0.007	0.085	0.004	0.228	0.025	0.214	0.029	0.725	0.014	0.443	0.035	NA	NA
10	10	200	60	0.3	5	0.585	0.043	0.088	0.003	0.063	0.004	0.225	0.026	0.208	0.026	0.717	0.015	0.428	0.035	NA	NA
1	1	200	60	0.05	2	0.292	0.031	0.029	0.024	0.070	0.013	0.055	0.040	0.054	0.046	0.935	0.031	0.206	0.029	0.022	0.520
1	2	200	60	0.05	2	0.309	0.027	0.030	0.026	0.067	0.013	0.052	0.041	0.052	0.048	0.940	0.032	0.159	0.027	0.030	0.431
1	3	200	60	0.05	2	0.295	0.033	0.029	0.027	0.070	0.014	0.036	0.038	0.036	0.043	0.964	0.029	0.186	0.031	0.024	0.329
1	4	200	60	0.05	2	0.285	0.031	0.030	0.024	0.076	0.013	0.040	0.040	0.040	0.046	0.958	0.031	0.198	0.029	0.031	0.326
1	5	200	60	0.05	2	0.284	0.033	0.032	0.026	0.080	0.013	0.042	0.038	0.042	0.043	0.955	0.029	0.174	0.031	0.027	0.364
1	6	200	60	0.05	2	0.260	0.031	0.030	0.026	0.084	0.013	0.043	0.041	0.043	0.046	0.954	0.031	0.188	0.029	0.023	0.402
1	7	200	60	0.05	2	0.266	0.027	0.028	0.026	0.076	0.013	0.052	0.038	0.052	0.043	0.940	0.032	0.177	0.027	0.048	0.337
1	8	200	60	0.05	2	0.304	0.033	0.032	0.026	0.074	0.014	0.056	0.040	0.058	0.048	0.976	0.029	0.203	0.031	0.027	0.489
1	9	200	60	0.05	2	0.246	0.031	0.026	0.024	0.078	0.013	0.093	0.041	0.095	0.046	0.927	0.031	0.183	0.029	0.038	0.687
1	10	200	60	0.05	2	0.265	0.027	0.029	0.026	0.080	0.013	0.075	0.038	0.077	0.043	0.948	0.029	0.184	0.031	0.035	0.576
5	1	200	60	0.05	2	0.270	0.065	0.019	0.021	0.064	0.012	0.048	0.033	0.048	0.034	0.943	0.023	0.262	0.050	0.017	0.495
5	2	200	60	0.05	2	0.229	0.050	0.016	0.026	0.061	0.013	0.045	0.031	0.045	0.032	0.948	0.024	0.112	0.047	0.012	0.513
5	3	200	60	0.05	2	0.208	0.047	0.022	0.021	0.064	0.014	0.029	0.034	0.029	0.036	0.972	0.026	0.125	0.047	0.023	0.271
5	4	200	60	0.05	2	0.256	0.065	0.019	0.021	0.070	0.013	0.033	0.034	0.033	0.036	0.966	0.023	0.204	0.047	0.016	0.348
5	5	200	60	0.05	2	0.213	0.047	0.018	0.021	0.074	0.013	0.035	0.035	0.035	0.035	0.963	0.025	0.200	0.047	0.015	0.378
5	6	200	60	0.05	2	0.196	0.050	0.029	0.021	0.078	0.013	0.036	0.033	0.036	0.034	0.962	0.025	0.212	0.047	0.010	0.429
5	7	200	60	0.05	2	0.271	0.047	0.021	0.021	0.070	0.013	0.045	0.031	0.045	0.032	0.948	0.023	0.112	0.047	0.017	0.467
5	8	200	60	0.05	2	0.231	0.065	0.018	0.021	0.068	0.013	0.049	0.034	0.051	0.036	0.984	0.024	0.208	0.047	0.017	0.509
5	9	200	60	0.05	2	0.209	0.050	0.024	0.021	0.072	0.013	0.046	0.034	0.047	0.036	0.965	0.024	0.204	0.047	0.009	1.057
5	10	200	60	0.05	2	0.250	0.047	0.023	0.021	0.074	0.013	0.048	0.035	0.048	0.035	0.956	0.026	0.193	0.047	0.019	0.680
10	1	200	60	0.05	2	0.237	0.062	0.014	0.017	0.062	0.013	0.047	0.034	0.047	0.036	0.960	0.023	0.210	0.051	0.018	0.479
10	2	200	60	0.05	2	0.208	0.045	0.016	0.015	0.059	0.014	0.052	0.035	0.053	0.038	0.968	0.025	0.185	0.047	0.015	0.778
10	3	200	60	0.05	2	0.173	0.041	0.013	0.011	0.062	0.014	0.044	0.033	0.045	0.035	0.975	0.025	0.108	0.044	0.011	0.517
10	4	200	60	0.05	2	0.181	0.047	0.015	0.015	0.068	0.014	0.064	0.033	0.067	0.034	0.979	0.023	0.181	0.050	0.012	0.731
10	5	200	60	0.05	2	0.174	0.065	0.015	0.011	0.072	0.014	0.057	0.031	0.059	0.032	0.980	0.024	0.152	0.047	0.016	0.603
10	6	200	60	0.05	2	0.160	0.050	0.015	0.017	0.076	0.014	0.056	0.034	0.058	0.036	0.985	0.023	0.153	0.047	0.016	0.588
10	7	200	60	0.05	2	0.174	0.047	0.014	0.017	0.068	0.014	0.058	0.031	0.060	0.036	0.982	0.025	0.158	0.051	0.013	0.650
10	8	200	60	0.05	2	0.182	0.062	0.015	0.017	0.066	0.014	0.064	0.034	0.066	0.035	0.972	0.025	0.169	0.047	0.014	0.713
10	9	200	60	0.05	2	0.175	0.045	0.015	0.015	0.070	0.014	0.058	0.034	0.061	0.038	0.983	0.023	0.145	0.044	0.017	0.605
10	10	200	60	0.05	2	0.189	0.041	0.017	0.011	0.072	0.014	0.055	0.035	0.058	0.035	0.989	0.024	0.122	0.050	0.015	0.592
1	1	200	60	0.5	2	0.928	0.031	0.346	0.024	0.054	0.013	0.490	0.040	0.530	0.046	0.552	0.031	0.923	0.029	0.200	1.117
1	2	200	60	0.5	2	0.953	0.027	0.360	0.026	0.036	0.013	0.487	0.041	0.525	0.048	0.553	0.032	0.876	0.027	0.242	0.941
1	3	200	60	0.5	2	0.929	0.033	0.352	0.027	0.054	0.014	0.471	0.038	0.501	0.043	0.563	0.029	0.903	0.031	0.259	0.858
1	4	200	60	0.5	2	0.923	0.031	0.364	0.024	0.061	0.013	0.475	0.040	0.507	0.046	0.560	0.031	0.915	0.029	0.236	0.939
1	5	200	60	0.5	2	0.921	0.033	0.380	0.026	0.065	0.013	0.477	0.038	0.510	0.043	0.559	0.029	0.891	0.031	0.209	1.048
1	6	200	60	0.5	2	0.902	0.031	0.354	0.026	0.077	0.013	0.478	0.041	0.511	0.046	0.558	0.031	0.905	0.029	0.233	0.954
1	7	200	60	0.5	2	0.906	0.027	0.330	0.026	0.068	0.013	0.487	0.038	0.525	0.043	0.553	0.032	0.894	0.027	0.212	1.054
1	8	200	60	0.5	2	0.908	0.033	0.388	0.026	0.079	0.014	0.491	0.040	0.489	0.048	0.507	0.029	0.920	0.031	0.232	0.985
1	9	200	60	0.5	2	0.983	0.031	0.306	0.024	0.011	0.013	0.528	0.041	0.538	0.046	0.481	0.031	0.900	0.029	0.224	1.090
1	10	200	60	0.5	2	0.971	0.027	0.346	0.026	0.021	0.013	0.510	0.038	0.517	0.043	0.497	0.029	0.901	0.031	0.228	1.039
5	1	200	60	0.5	2	0.710	0.065	0.226	0.021	0.185	0.012	0.483	0.033	0.522	0.034	0.559	0.023	0.779	0.050	0.128	1.589
5	2	200	60	0.5	2	0.653	0.050	0.188	0.026	0.200	0.013	0.480	0.031	0.517	0.032	0.560	0.024	0.629	0.047	0.113	1.740
5	3	200	60	0.5	2	0.820	0.047	0.268	0.021	0.118	0.014	0.464	0.034	0.493	0.036	0.570	0.026	0.642	0.047	0.110	1.716
5	4	200	60	0.5	2	0.762	0.065	0.232	0.021	0.145	0.013	0.468	0.034	0.499	0.036	0.567	0.023	0.721	0.047	0.120	1.624
5	5	200	60	0.5	2	0.668	0.047	0.220	0.021	0.219	0.013	0.470	0.035	0.502	0.035	0.566	0.025	0.717	0.047	0.139	1.445
5	6	200	60	0.5	2	0.759	0.050	0.344	0.021	0.218	0.013	0.471	0.033	0.503	0.034	0.565	0.025	0.729	0.047	0.131	1.525
5	7	200	60	0.5	2	0.691	0.047	0.256	0.021	0.229	0.013	0.480	0.031	0.517	0.032	0.560	0.023	0.629	0.047	0.118	1.680
5	8	200	60	0.5	2	0.974	0.065	0.210	0.021	0.011	0.013	0.484	0.034	0.481	0.036	0.513	0.024	0.725	0.047	0.144	1.453
5	9	200	60	0.5	2	0.914	0.050	0.284	0.021	0.053	0.013	0.521	0.034	0.530	0.036	0.487	0.024	0.721	0.047	0.113	1.887
5	10	200	60	0.5	2	0.811	0.047	0.278	0.021	0.130	0.013	0.503	0.035	0.509	0.035	0.503	0.026	0.710	0.047	0.116	1.782
10	1	200	60	0.5	2	0.965	0.062	0.170	0.017	0.012	0.013	0.482	0.034	0.469	0.036	0.504	0.023	0.927	0.051	0.099	1.930
10	2	200	60	0.5	2	0.863	0.045	0.186	0.015	0.059	0.014	0.507	0.035	0.497	0.038	0.483	0.025	0.902	0.047	0.107	1.915
10	3	200	60	0.5	2	0.943	0.041	0.156	0.011	0.019	0.014	0.479	0.033	0.470	0.035	0.511	0.025	0.825	0.044	0.106	1.822
10	4	200	60	0.5	2	0.884	0.047	0.180	0.015	0.047	0.014	0.499	0.033	0.486	0.034	0.488	0.023	0.898	0.050	0.097	2.030
10	5	200	60	0.5	2	0.878	0.065	0.182	0.011	0.051	0.014	0.492	0.031	0.485	0.032	0.501	0.024	0.869	0.047	0.101	1.944
10	6	200	60	0.5	2	0.891	0.050	0.174	0.017	0.043	0.014	0.491	0.034	0.480	0.036	0.498	0.023	0.870	0.047	0.101	1.934
10	7	200	60	0.5	2	0.905	0.047	0.172	0.017	0.036	0.014	0.493	0.031	0.483	0.036	0.497	0.025	0.875	0.051	0.101	1.940
10	8	200	60	0.5	2	0.903	0.062	0.176	0.017	0.038	0.014	0.499	0.034	0.493	0.035	0.495	0.025	0.886	0.047	0.106	1.896
10	9	200	60	0.5	2	0.902	0.045	0.178	0.015	0.039	0.014	0.493	0.034	0.482	0.038	0.496	0.023	0.862	0.044	0.116	1.754
10	10	200	60	0.5	2	0.903	0.041	0.202	0.011	0.043	0.014	0.490	0.035	0.476	0.035	0.495	0.024	0.839	0.050	0.102	1.922
