# Supplementary material for: Estimating heritability using family-pooled phenotypic and genotypic data: a simulation study applied to aquaculture
Source: Heredity (Edinb). 2022 Jan 31;128(3):178–86. doi: 10.1038/s41437-022-00502-8 (PMC8897491; doi:10.1038/s41437-022-00502-8)
Supplement: Supplementary file 3 — S3_Heritability of family means (pool), repeatability (t) and their respective standard erros (SE). The results are for two-pool Scenario from S3 for trait heritability of 0.3. [file 41437_2022_502_MOESM3_ESM.pdf]

Supplementary Table S3. Heritability of family means (pool), repeatability (t) and their respective standard erros (SE). The results are for two-pool Scenario from S3 for trait heritability of 0.3.

Generation	Replicate	Pool Size	h^2 (pool)	SE	t	SE
1	1	12	0.813	0.069	0.659	0.083
1	2	12	0.835	0.065	0.635	0.088
1	3	12	0.828	0.065	0.720	0.070
1	4	12	0.819	0.065	0.665	0.089
1	5	12	0.841	0.065	0.641	0.094
1	6	12	0.834	0.070	0.726	0.076
1	7	12	0.838	0.065	0.638	0.091
1	8	12	0.831	0.065	0.723	0.073
1	9	12	0.822	0.065	0.668	0.092
1	10	12	0.844	0.065	0.644	0.097
5	1	12	0.000	0.000	0.286	0.132
5	2	12	0.548	0.122	0.390	0.123
5	3	12	0.407	0.160	0.504	0.109
5	4	12	0.413	0.110	0.292	0.130
5	5	12	0.000	0.000	0.396	0.120
5	6	12	0.554	0.150	0.510	0.110
5	7	12	0.413	0.110	0.393	0.120
5	8	12	0.410	0.150	0.507	0.110
5	9	12	0.416	0.110	0.295	0.130
5	10	12	0.000	0.000	0.399	0.120
10	1	12	0.284	0.154	0.143	0.141
10	2	12	0.648	0.112	0.530	0.104
10	3	12	0.256	0.145	0.253	0.135
10	4	12	0.290	0.130	0.149	0.130
10	5	12	0.654	0.120	0.536	0.130
10	6	12	0.262	0.120	0.259	0.140
10	7	12	0.287	0.110	0.256	0.140
10	8	12	0.651	0.110	0.152	0.140
10	9	12	0.259	0.110	0.539	0.142
10	10	12	0.293	0.100	0.262	0.144
1	1	30	0.872	0.043	0.850	0.040
1	2	30	0.834	0.052	0.854	0.039
1	3	30	0.896	0.037	0.872	0.034
1	4	30	0.880	0.038	0.858	0.032
1	5	30	0.842	0.035	0.862	0.029
1	6	30	0.904	0.032	0.880	0.039
1	7	30	0.837	0.029	0.857	0.040
1	8	30	0.899	0.026	0.875	0.036
1	9	30	0.883	0.023	0.861	0.035
1	10	30	0.845	0.020	0.865	0.035
5	1	30	0.619	0.130	0.607	0.090
5	2	30	0.763	0.073	0.722	0.069

5	3	30	0.807	0.065	0.779	0.056
5	4	30	0.627	0.057	0.615	0.043
5	5	30	0.771	0.049	0.730	0.030
5	6	30	0.815	0.130	0.787	0.090
5	7	30	0.766	0.073	0.725	0.069
5	8	30	0.810	0.102	0.782	0.057
5	9	30	0.630	0.110	0.618	0.055
5	10	30	0.774	0.119	0.733	0.054
10	1	30	0.276	0.148	0.533	0.102
10	2	30	0.802	0.069	0.726	0.068
10	3	30	0.798	0.070	0.523	0.104
10	4	30	0.284	0.071	0.541	0.093
10	5	30	0.810	0.072	0.734	0.094
10	6	30	0.806	0.119	0.531	0.095
10	7	30	0.805	0.148	0.729	0.096
10	8	30	0.801	0.114	0.526	0.097
10	9	30	0.287	0.118	0.544	0.098
10	10	30	0.813	0.122	0.737	0.099
1	1	60	0.911	0.029	0.919	0.022
1	2	60	0.919	0.026	0.917	0.023
1	3	60	0.951	0.018	0.908	0.025
1	4	60	0.919	0.037	0.927	0.030
1	5	60	0.927	0.034	0.925	0.031
1	6	60	0.959	0.026	0.916	0.033
1	7	60	0.922	0.029	0.920	0.026
1	8	60	0.954	0.021	0.911	0.028
1	9	60	0.922	0.040	0.930	0.033
1	10	60	0.930	0.037	0.928	0.034
5	1	60	0.866	0.047	0.770	0.058
5	2	60	0.865	0.042	0.829	0.045
5	3	60	0.893	0.036	0.725	0.068
5	4	60	0.873	0.054	0.777	0.065
5	5	60	0.872	0.049	0.836	0.052
5	6	60	0.900	0.043	0.732	0.075
5	7	60	0.867	0.044	0.831	0.047
5	8	60	0.895	0.038	0.727	0.070
5	9	60	0.875	0.056	0.779	0.067
5	10	60	0.874	0.051	0.838	0.054
10	1	60	0.642	0.112	0.573	0.096
10	2	60	0.891	0.035	0.858	0.038
10	3	60	0.810	0.063	0.704	0.072
10	4	60	0.651	0.121	0.582	0.105
10	5	60	0.900	0.044	0.867	0.047
10	6	60	0.819	0.072	0.713	0.081
10	7	60	0.896	0.040	0.863	0.043
10	8	60	0.815	0.068	0.709	0.077
10	9	60	0.656	0.126	0.587	0.110

10	10	60	0.905	0.049	0.872	0.052
1	1	100	0.971	0.011	0.964	0.010
1	2	100	0.940	0.019	0.933	0.019
1	3	100	0.963	0.013	0.955	0.013
1	4	100	0.975	0.015	0.968	0.014
1	5	100	0.944	0.023	0.937	0.023
1	6	100	0.967	0.017	0.959	0.017
1	7	100	0.942	0.022	0.936	0.020
1	8	100	0.965	0.016	0.958	0.014
1	9	100	0.977	0.018	0.971	0.015
1	10	100	0.946	0.026	0.940	0.024
5	1	100	0.930	0.023	0.831	0.044
5	2	100	0.918	0.025	0.895	0.028
5	3	100	0.952	0.016	0.902	0.027
5	4	100	0.934	0.027	0.835	0.048
5	5	100	0.922	0.029	0.899	0.032
5	6	100	0.956	0.020	0.906	0.031
5	7	100	0.920	0.028	0.898	0.029
5	8	100	0.954	0.019	0.905	0.028
5	9	100	0.936	0.030	0.838	0.049
5	10	100	0.924	0.032	0.902	0.033
10	1	100	0.771	0.077	0.710	0.071
10	2	100	0.939	0.020	0.888	0.030
10	3	100	0.897	0.035	0.802	0.051
10	4	100	0.775	0.081	0.714	0.075
10	5	100	0.943	0.024	0.892	0.034
10	6	100	0.901	0.039	0.806	0.055
10	7	100	0.941	0.023	0.891	0.031
10	8	100	0.899	0.038	0.805	0.052
10	9	100	0.777	0.084	0.717	0.076
10	10	100	0.945	0.027	0.895	0.035
